# Improvements in the HIV care continuum needed to meaningfully reduce HIV incidence among men who have sex with men in Baltimore, US: a modelling study for HPTN 078

**DOI:** 10.1002/jia2.25246

**Published:** 2019-03-14

**Authors:** Kate M Mitchell, Brooke Hoots, Dobromir Dimitrov, Danielle German, Colin Flynn, Jason E Farley, Marcy Gelman, James P Hughes, Deborah Donnell, Adeola Adeyeye, Robert H Remien, Chris Beyrer, Gabriela Paz‐Bailey, Marie‐Claude Boily

**Affiliations:** ^1^ Department of Infectious Disease Epidemiology Imperial College London HPTN Modelling Centre London UK; ^2^ Division of STD Prevention Centers for Disease Control and Prevention Atlanta GA USA; ^3^ Vaccine and Infectious Disease Division Fred Hutchinson Cancer Research Center Seattle WA USA; ^4^ Department of Health, Behavior and Society Johns Hopkins Bloomberg School of Public Health Baltimore MD USA; ^5^ Center for HIV Surveillance, Epidemiology and Evaluation Maryland Department of Health Baltimore MD USA; ^6^ Department of Community‐Public Health Johns Hopkins University School of Nursing Baltimore MD USA; ^7^ The Fenway Institute Fenway Health Boston MA USA; ^8^ Department of Biostatistics University of Washington Seattle WA USA; ^9^ Division of AIDS, NIAID National Institutes of Health Washington DC USA; ^10^ HIV Center for Clinical and Behavioral Studies NY State Psychiatric Institute New York NY USA; ^11^ Department of Psychiatry Columbia University New York NY USA; ^12^ Department of Epidemiology Johns Hopkins Bloomberg School of Public Health Baltimore MD USA; ^13^ Division of HIV/AIDS Prevention Centers for Disease Control and Prevention Atlanta GA USA

**Keywords:** HIV infections, incidence, homosexuality, male, models, theoretical, United States, forecasting

## Abstract

**Introduction:**

HIV prevalence is high among men who have sex with men (MSM) in Baltimore, Maryland, United States, and the levels of viral suppression among HIV‐positive MSM are relatively low. The HIV Prevention Trials Network 078 trial seeks to increase the levels of viral suppression among US MSM by increasing the rates of diagnosis and linkage to care and treatment. We estimated the increases in viral suppression needed to reach different HIV incidence reduction targets, and the impact of meeting diagnosis and treatment targets.

**Methods:**

We used a mathematical model of HIV transmission among MSM from Baltimore, US, parameterised with behavioural data and fitted to HIV prevalence and care continuum data for Baltimore wherever possible, to project increases in viral suppression needed to reduce the HIV incidence rate among Baltimore MSM by 10, 20, 30 or 50% after 2, 5 and 10 years. We also projected HIV incidence reductions achieved if US national targets – 90% of people living with HIV (PLHIV) know their HIV serostatus, 90% of those diagnosed are retained in HIV medical care and 80% of those diagnosed are virally suppressed – or UNAIDS 90‐90‐90 targets (90% of PLHIV know their status, 90% of those diagnosed receive antiretroviral therapy (ART), 90% of those receiving ART are virally suppressed) are each met by 2020.

**Results:**

To reduce the HIV incidence rate by 20% and 50% after five years (compared with the base‐case at the same time point), the proportion of all HIV‐positive MSM who are virally suppressed must increase above 2015 levels by a median 13 percentage points (95% uncertainty interval 9 to 16 percentage points) from median 49% to 60%, and 27 percentage points (22 to 35) from 49% to 75% respectively. Meeting all three US or 90‐90‐90 UNAIDS targets results in a 48% (31% to 63%) and 51% (38% to 65%) HIV incidence rate reduction in 2020 respectively.

**Conclusions:**

Substantial improvements in levels of viral suppression will be needed to achieve significant incidence reductions among MSM in Baltimore, and to meet 2020 US and UNAIDS targets. Future modelling studies should additionally consider the impact of pre‐exposure prophylaxis for MSM.

## 1 Introduction

HIV incidence and prevalence remain high among gay, bisexual and other men who have sex with men (MSM) in the United States (US) 1, 2, 3. Baltimore, Maryland (population 621,000 in 2010) is one of the cities with the highest HIV prevalence among MSM in the US, reaching 43% in the Centers for Disease Control's (CDC) National HIV Behavioral Surveillance (NHBS) survey conducted in 2011, and falling to 30% in 2014 3, 4. Only an estimated 40% of diagnosed HIV‐positive MSM in Baltimore were virally suppressed in 2015 (54% had a viral load test, of whom 74% were suppressed 5).

As well as improving the survival and health of HIV‐positive persons 6, receiving antiretroviral therapy (ART) for HIV/AIDS and being virally suppressed greatly reduce the risk of transmitting HIV to sexual partners 7, 8. Higher ART coverage is associated with reduced population‐level HIV incidence 9.

Targets designed to improve viral suppression levels and reduce HIV incidence by 2020 have been put forward for the US 10, and by UNAIDS 11. US targets include reducing the total number of new HIV diagnoses in 2020 by at least 25% compared with 2010 (US new diagnoses target), while increasing the percentage of people living with HIV (PLHIV) who know their serostatus to 90%, the percentage of those diagnosed who are in care to 90%, and the percentage of diagnosed persons who are virally suppressed to 80% by 2020 (US continuum targets) 10. The UNAIDS continuum “90‐90‐90” targets aim to increase the percentages of PLHIV who know their HIV status, diagnosed PLHIV receiving ART, and PLHIV receiving ART who are virally suppressed each to 90% by 2020 11. Note that those receiving ART (UNAIDS target 2) are a subset of those in care (US target 2).

The HIV Prevention Trials Network (HPTN) 078 trial, ongoing since March 2016 among MSM in four US cities including Baltimore, aims to increase diagnosis, linkage to care and ART use for HIV‐positive MSM (https://www.hptn.org/research/studies/154). The primary trial objectives are to assess the ability of deep‐chain respondent‐driven sampling (using long multi‐wave recruitment chains) to identify non‐virally suppressed HIV‐positive MSM, and to test the efficacy of an enhanced case‐manager intervention package for helping participants to achieve and sustain viral suppression two years after being assigned to the intervention. The trial protocol includes two modelling objectives. The first objective is addressed in this analysis, prior to obtaining trial results, using the best available data. The second objective will be addressed after trial completion, using the same model with trial data to assess the impact of the study intervention on HIV incidence in the wider MSM population in each of the four cities.

In this analysis, we developed and used a mathematical model of HIV transmission and treatment for US MSM, parameterized for Baltimore, to project (1) the increase in viral suppression needed to reduce the HIV incidence rate among MSM by 10, 20, 30 or 50% after 2, 5 or 10 years (representing reduction targets that may be chosen by health departments or clinical trials 10, 12, 13, 14), (2) reduction in HIV incidence achieved if US or UNAIDS targets are met and (3) potential for HIV elimination among MSM in Baltimore.

## 2 Methods

### 2.1 Model structure

We developed a deterministic, compartmental dynamic model of HIV transmission for sexually active US MSM, taking into account demography, HIV natural history (disease progression) and interventions (Figures S1,S2,S3).

The model divides the MSM population by age (young: 18 to 24, older: >24 years old 15), race (black, white), infection status, set‐point viral load (SPVL), stage of disease progression by CD4 T‐cell count and HIV care continuum stage.

Uninfected MSM join the modelled population through ageing in, sexual debut, or migration, and MSM leave through HIV‐related and ‐unrelated mortality, migration or ceasing to attend surveillance venues (such as bars, clubs and cafés).

Following HIV acquisition, modelled individuals move into the high‐infectivity acute stage, and then into one of 16 chronic infection states defined by SPVL and CD4 count 16. Those not on ART, and those on ART but non‐adherent, move through progressively lower CD4 states 16; those adherent to ART do not. Increases in CD4 count for those adherent to ART are not explicitly modelled, but survival on ART is modelled as a function of initial CD4 count.

In the model, any HIV‐positive MSM can be diagnosed and start ART once symptomatic. A fraction are assumed to never routinely test for HIV. Other HIV‐positive MSM routinely test (at age‐ and race‐specific rates) and leave the undiagnosed state, with a proportion assumed to link directly into care, while the remainder are diagnosed but unlinked, and may subsequently move into care. Those in care may dropout into the diagnosed unlinked state. Those in care may initiate ART, with changes over time in ART eligibility (related to CD4 count) explicitly modelled following past CDC guidelines (Table S1). A proportion are adherent, achieving full viral suppression following a short period of partial suppression; the non‐adherent remainder do not achieve viral suppression (loss and regain of viral suppression are not explicitly modelled, due to a lack of data). Those on ART can drop out of care, and may subsequently re‐initiate ART following care re‐engagement.

Modelled HIV transmission occurs in main, casual and commercial sexual partnerships with men, which differ in number of sex acts and condom use. The age‐ and race‐specific per‐capita infection rate depends upon numbers of new partners/year, sex acts/partnership, transmission probability/sex act, circumcision status, condom use, and HIV prevalence, viral load, infection stage and viral suppression among sexual partners.

The model was expressed as a set of differential equations, solved numerically in C++ using a variable‐stepsize eighth‐order Runge‐Kutta method 17. See Supporting Information for equations and further details.

### 2.2 Model calibration

The model was parameterized using data from multiple sources (see section 2.3.5), and fitted to Baltimore MSM data in four steps: (1) we defined plausible ranges of values for all parameters in five domains: demography, sexual behaviour, HIV natural history and infectivity, care continuum and intervention efficacies, (2) sampled all parameter ranges one million times using Latin Hypercube sampling 18 and, with each parameter set, simulated the HIV epidemic from 1984 until 2016, (3) retained parameter sets for which model predictions simultaneously agreed with demographic, epidemiological and care continuum fitting outcome estimates before 2014 (Tables 1 and S2), (4) compared model predictions for these retained parameter sets with 2014 to 2017 demographic, epidemiological and care continuum validation data.

**Table 1:** Summary of key sexual behaviour, care continuum and intervention efficacy parameters and fitting outcomes

Parameters	Range	Source
Sexual behaviour parameters		
Average number of new main anal sex partners per year 2011^a^		
18‐ to 24‐year‐old black MSM	0.58 to 0.8^b^	Baltimore NHBS
>24‐year‐old black MSM	0.36 to 0.57^b^	
18‐ to 24‐year‐old white MSM	0.08 to 0.37^b^	
>24‐year‐old white MSM	0.11 to 0.21^b^	
Average number of new casual anal sex partners per year 2011^a^		
18‐ to 24‐year‐old black MSM	1.54 to 2.09^b^	Baltimore NHBS
>24‐year‐old black MSM	0.81 to 1.24^b^	
18‐ to 24‐year‐old white MSM	0.05 to 0.93^b^	
>24‐year‐old white MSM	0.28 to 1.07^b^	
Average number of new commercial anal sex partners per year 2011^a^		
18‐ to 24‐year‐old black MSM	0 to 1.36^b^	Baltimore NHBS
>24‐year‐old black MSM	0.15 to 0.85^b^	
18‐ to 24‐year‐old white MSM	0 to 0.28^b^	
>24‐year‐old white MSM	0 to 0.07^b^	
Percentage of sex acts in which condom used 2011^a^		
Main partnerships, both partners black	47 to 67	Baltimore NHBS
Main partnerships, either partner white	30 to 39	
Casual partnerships (any race partner)	63 to 72	
Commercial partnerships (any race partner)	21 to 78	
Care continuum parameters		
Percentage of undiagnosed MSM testing for HIV per year, 2011^a^		
18‐ to 24‐year‐old black MSM	63.8 to 95.0	Baltimore NHBS
>24‐year‐old black MSM	50.0 to 70.2	
18‐ to 24‐year‐old white MSM	32.1 to 82.3	
>24‐year‐old white MSM	32.7 to 69.7	
Percentage of white MSM testing positive for HIV who are immediately linked to care, 2008^a^	67 to 85	Maryland DH Baltimore data, national US MSM estimates (data on linkage within three months of diagnosis) 24, 35
Ratio of percentage of black MSM linking to care immediately compared to white MSM	0.84 to 1.5	
Rate of linkage to care per year for those not linking immediately or dropped out, white MSM, 2008^a^	0 to 0.5	Fitted
Ratio of rate of linkage to care for black MSM compared to white MSM	0.84 to 2	National US MSM estimates 24, 35
Rate of initiation onto ART from care per year, when meeting CD4 criteria, all years; permitted to differ by race	0.5 to 4	CD4 testing every 3 to 6 months (national guidelines), 80 to 90% acceptance 36
Percentage of white MSM initiating ART who are adherent (achieve viral suppression), all years	73 to 99	US studies (multiple sites) 24, 37, 38, 39
Ratio of percentage adherent to ART black: white MSM	0.82 to 1	US studies (multiple sites) 24, 37, 38, 39
Rate of dropout from ART per year first two years on ART, all years	0.06 to 0.13	US studies (multiple sites) 40, 41, 42, 43
Ratio of dropout from ART third+ years on ART: first two years on ART	0.5 to 1.0	US ART cohorts (multiple sites) 44
Ratio of rate of dropout from care: rate of dropout from ART, black MSM	1 to 7	US studies (multiple sites) 41, 42, 45, 46
Ratio of dropout from care for white: black MSM	0.46 to 3	US studies (multiple sites) 41, 42, 45
Intervention efficacies		
Per‐sex‐act reduction in HIV acquisition risk due to correct condom use (%)	58 to 79	US MSM estimates (multiple sites) 47
Per‐sex‐act reduction in HIV acquisition risk due to male circumcision (%)	12 to 23	Assuming efficacy only for insertive anal sex
Per‐sex‐act reduction in HIV transmission risk due to being on ART and fully suppressed (%)	99 to 100	European MSM estimates (multiple countries) 8

Fitting outcomes	Range	Source
Demography indicators		
MSM population size 2010	4270 to 8765	Baltimore census and national estimates of % men who are MSM
Percentage MSM aged 18 to 24		
2010	11 to 21	Baltimore census (all males) ± 5 pp
2011	22 to 42	Baltimore NHBS (95% CI)
Percentage MSM black		
2010	64 to 74	Baltimore census (all males) ± 5 pp
2011	72 to 92	Baltimore NHBS (95% CI)
Epidemiological indicators		
HIV prevalence 2011 (%)		
18‐ to 24‐year‐old black MSM	32 to 48	Baltimore NHBS (95% CI)
>24‐year‐old black MSM	39 to 56	
18‐ to 24‐year‐old white MSM	ND	
>24‐year‐old white MSM	12 to 30	
Care continuum indicators		
Percentage of HIV‐positive MSM diagnosed, 2012	72 to 81	CDC data for Maryland (95% CI) 4
Percentage of HIV‐positive MSM on ART, 2011		
Overall	48 to 63	Baltimore NHBS plasma testing (95% CI)
Black	44 to 59	
Percentage of diagnosed MSM in care, 2013		
Black	57 to 70	Maryland DH Baltimore data ± 5 pp (range 2012 to 2013)
White	45 to 59	
Percentage of diagnosed MSM virally suppressed, 2013		
Black	32 to 42	Maryland DH Baltimore data ± 5 pp
White	34 to 44	
Percentage of MSM on ART virally suppressed, 2010	75 to 90	National US MSM estimates (range) 23, 24

^a^Time‐varying parameters; final values reported here, earlier time‐trends described in Supporting Information and in Table S1; ^b^range calculated by taking the upper and lower bounds from the 95% CI for the number of anal sex partners reported in the past 12 months, and adjusted for the proportion of partners who are reported to be “new.” Full details in Tables S1 and S2. For parameters, range is full range of plausible values explored; for fitting outcomes, range gives bounds within which model outcomes must fall to be retained. ND, no data; MSM, men who have sex with men; NHBS, National HIV Behavioural Surveillance; DH, Department of Health; US, United States; ART, antiretroviral therapy; CDC, Centers for Disease Control.

#### 2.2.1 Data sources – model parameters and fitting outcomes

Local data sources informed demography, sexual behaviour and care continuum parameters wherever possible 3, 5, 19, 20, 21. HIV natural history, infectivity and intervention efficacy parameters were drawn from published literature, on MSM and/or Western populations where possible.

NHBS were serial cross‐sectional studies conducted among MSM in Baltimore 3, 19, 20. Data collection methods have been described previously 22. Briefly, MSM aged over 18 were recruited using venue‐based, time‐space sampling, administered a face‐to‐face behavioural interview and HIV testing. NHBS data collection was approved by the Maryland Department of Health (DH), Johns Hopkins Bloomberg School of Public Health Institutional Review Boards, and CDC. All those with a valid HIV test result reporting sex with another man in the last 12 months were included in the current analysis. Individual‐level data were analysed using SAS 9.4 (SAS Institute, Cary, NC, USA). Clusters (at the recruitment venue level) were taken into account using the SAS surveymeans and surveyfreq procedures.

Data for MSM from the Johns Hopkins HIV cohort 21 and data from NA‐ACCORD from Fenway electronic monitoring data were used to estimate median time to viral suppression stratified by initial viral load.

Estimates and sources for key behaviour and care continuum parameters are presented in Table 1, and for all parameters in Table S1. See Supporting Information for further details.

Demographic, epidemiological and care continuum fitting (pre‐2014) or validation (2014 to 2017) outcomes were based on local data sources, plus national estimates for levels of viral suppression among those on ART 23, 24. Data informing ART coverage came from a Baltimore MSM NHBS sub‐study (2008 to 2014), which tested stored sera for antiretrovirals 25 among participants who consented to storage for future testing. Fitting outcomes and sources are summarized in Table 1, full details in Table S2.

### 2.3 Plan of analysis

#### 2.3.1 Addressing uncertainties across data sources

Different data sources provided incompatible estimates – that is if the model was fitted to one data source, it did not fit the other – for three fitting outcomes. To address this uncertainty, we defined six fitting assumptions, using different ranges for relevant input parameters (Table S1) in order to fit to different data sources (Table S2). We fitted to two age/race distribution assumptions, from either (1) NHBS or (2) census data. We used two diagnosis assumptions, either (3) using NHBS testing rate parameters or (4) fitting the percentage of diagnosed MSM to Maryland CDC estimates. We defined two care continuum assumptions, fitting to (5) NHBS ART or (6) DH continuum data (further details in Supporting Information). Using all combinations of these fitting assumptions gave eight groups of model fits.

#### 2.3.2 Base‐case scenario

Each retained parameter set was used to project the HIV epidemic from 1984 to 2036 (to allow examination of elimination over a 20 year period after 2016), in a base‐case scenario assuming rates of HIV testing, linkage to care, ART initiation, adherence and dropout remain at 2015 levels until 2036.

#### 2.3.3 Intervention scenarios

To project (1) required increases in viral suppression to reach incidence reduction targets and (2) HIV incidence reductions if US and UNAIDS targets are met, we explored different improved care continuum parameters, introduced in 2016 (as an instantaneous step change). We used Latin Hypercube sampling to select 1600 combinations of improved continuum parameter values sampled between 2015 levels and most optimistic values (in brackets): percentage HIV tested (98% tested/year), percentage of tested linked (100% link directly into care after diagnosis), rate of linkage into care (4/year/person among those diagnosed but unlinked (i.e. after average three months)), ART initiation rate (6/year/person (after average two months)), percentage ART adherent (100%), ART dropout rate (0%). All parameters were improved simultaneously in 2016 and maintained until 2036. We re‐ran each of the retained fitting parameter sets (from model calibration step 3) with each of these 1600 improved continuum parameter combinations in turn, and identified all combinations where the (1) HIV incidence rate reduction or (2) percentage at different care stages, fell within ±0.5 percentage points of pre‐defined targets (below). We used these to answer five questions.

#### 2.3.4 Question 1: What increase in viral suppression is needed to reach predefined HIV incidence reduction targets?

The targets were as follows: 10, 20, 30 or 50% relative reduction in HIV incidence rate 2, 5 or 10 years after care continuum parameters were improved at the start of 2016, compared with base‐case scenario at the same time‐points. Incidence is measured over a one‐year period.

For each intervention scenario meeting each target, we projected the absolute increase above 2015 levels in the percentage of HIV‐positive men virally suppressed at the time the target was met (Figure S4a,b). In the main analysis, results are pooled across the eight groups of model fits, to account for all of the uncertainty arising from different data sources. Results are also stratified by fitting assumption, to assess the sensitivity of results to these assumptions.

#### 2.3.5 Question 2: What care continuum improvements are needed to meet HIV incidence reduction targets?

We projected the expected levels of different care continuum indicators (diagnosed/in care/on ART/virally suppressed) when meeting incidence reduction targets after 2, 5 and 10 years, pooling results across the eight groups of model fits. We calculated Pearson correlation coefficients between the increase in each care continuum parameter above 2015 levels and HIV incidence rate reductions after five years across all intervention scenarios.

#### 2.3.6 Question 3: What reduction in HIV incidence will be achieved if US and UNAIDS targets are met in 2020?

1. US new diagnoses target: relative decline in new HIV diagnoses of 25% from 2010 to 2020.
2. US continuum targets: 90% of HIV‐positive diagnosed, 90% of diagnosed in care, 80% of diagnosed virally suppressed.
3. UNAIDS continuum targets: 90% of HIV‐positive diagnosed, 90% of diagnosed on treatment, 90% of treated virally suppressed.

We projected the relative reduction in HIV incidence rate compared with the base‐case scenario in 2020, across intervention scenarios meeting US and UNAIDS targets (Figure S4c,d).

#### 2.3.7 Question 4: What reduction in HIV incidence will be seen if the HPTN 078 trial target effect size is achieved?

The HPTN 078 trial was powered to detect an 18 percentage point difference in levels of viral suppression between men receiving a case‐manager intervention or standard of care, two years after enrolment. We projected the relative reduction in HIV incidence rate compared with the base‐case scenario when the percentage of diagnosed MSM virally suppressed is 18 percentage points higher in the intervention than the base‐case scenario after two years.

#### 2.3.8 Question 5: What is the potential for local HIV elimination?

We recorded how often HIV elimination (defined as HIV incidence <1 infection/1000 person‐years 26) occurred before 2036 in base‐case and intervention scenarios meeting incidence reduction, US, or UNAIDS targets.

## 3 Results

### 3.1 Model fits

**Figure 1:**
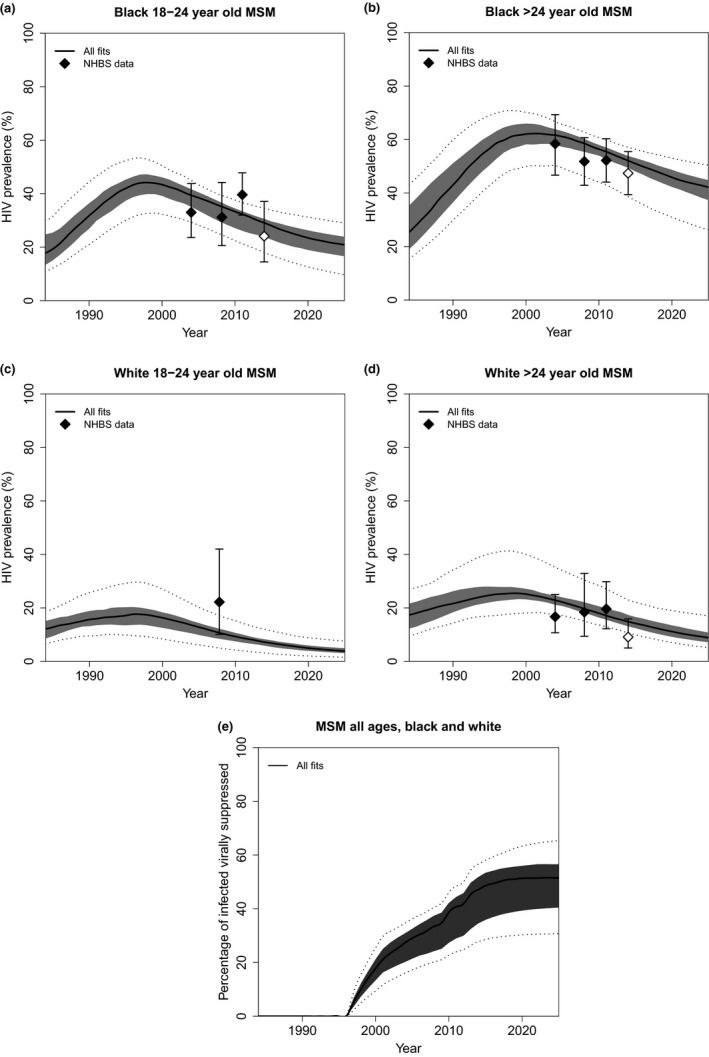
Model fits to available data for men who have sex with men (MSM) in Baltimore **(a, b, c, d)** HIV prevalence among young (18‐ to 24‐year‐old) black/older (>24‐year‐old) black/young white/older white MSM, **(e)** percentage of HIV‐positive MSM virally suppressed. Results are for all 169 fitting parameter combinations across eight groups of model fits (see section 2.3.1 “Addressing uncertainties across data sources” for more details). Results show median (thick lines), 25th to 75th percentile (dark shaded area), and 2.5th and 97.5th percentiles (dotted lines) across model fits. Points and error bars show the mean and 95% CI for HIV prevalence in National HIV Behavioural Surveillance data, measured with HIV testing (a,b,c,d). Data prior to 2014 (black points) were used for model fitting. Data from 2014 (white points) were used to validate model predictions.

Altogether, 169 unique parameter combinations, pooled across the eight groups of model fits, fitted available data. The model fitted age‐ and race‐specific HIV prevalence well, and adequately predicted 2014 HIV prevalence validation data, but slightly overestimated prevalence among older white MSM (Figure 1a,b,c,d).

Fits to NHBS ART coverage data gave higher estimates of percentages in care, on ART and virally suppressed (median 2015 estimates: 76%, 69% and 59% respectively), than fits to DH continuum data (median 2015 estimates: 63%, 49% and 41% respectively) (Figure S5f,g,h,i,j,k,l,m). The model predicted 2014 NHBS age/race and ART coverage and 2014 to 2017 DH care validation data well under the corresponding assumptions, but tended to underestimate 2014 to 2017 DH viral suppression (Figure S5a,b,c,d,f,g,h,j,k,l).

Viral suppression levels were projected to increase over time, reaching 49% (95% uncertainty interval (UI) 29% to 58%) of HIV‐positive MSM at the end of 2015 (Figure 1e).

### 3.2 Base‐case scenario

Under the base‐case scenario (keeping continuum parameters at 2015 levels), viral suppression levels continue to increase after 2015, by a median 2, 3 and 4 percentage points above 2015 levels after 2, 5 and 10 years respectively. Under the base‐case scenario, projected annual HIV incidence declines from 3.3 (UI 1.8 to 5.0)/100 py in 2015 to 3.0/100 py in 2017, 2.7/100 py in 2020 and 2.4/100 py in 2025.

### 3.3 Analysis

#### 3.3.1 Question 1: What increase in viral suppression is needed to reach predefined HIV incidence reduction targets?

**Figure 2:**
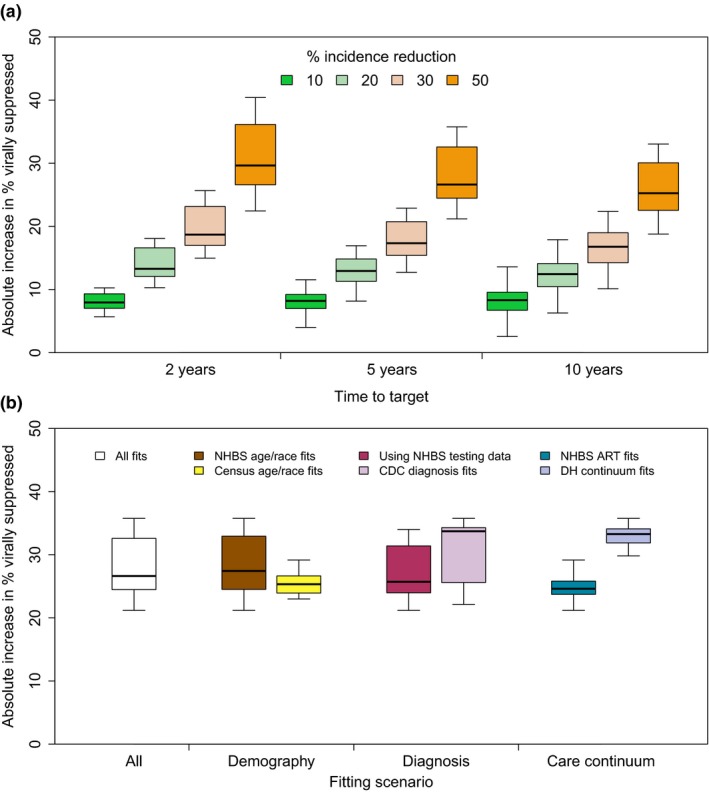
Required increase in viral suppression above initial 2015 level to meet different incidence reduction targets after different time periods (in comparison with the base‐case scenario at the same time point) **(a)** 10, 20, 30 or 50% incidence reduction after 2, 5 or 10 years (*x*‐axis), pooled across all fits; **(b)** 50% incidence reduction target after five years, across all fits, or stratified by: the two demography fitting assumptions (fitting to either the National HIV Behavioural Surveillance (NHBS) or census age/race distribution), two diagnosis fitting assumptions (using NHBS HIV testing rate parameters or fitting to Centres for Disease Control estimates for Maryland), or the two care continuum fitting assumptions, fitting to NHBS antiretroviral therapy coverage data or Department of Health continuum data (*x*‐axis). Note in (b) results for each stratification are pooled across the other stratifications used. Box and whiskers are calculated from mean values across included fits. The thick horizontal line, box and whiskers show the median, 25th to 75th percentiles, and minimum/maximum values.[Colour figure can be viewed at wileyonlinelibrary.com]

The annual HIV incidence rate is projected to be reduced by 20% (compared to the base‐case scenario at the same time point) after 2, 5 and 10 years if viral suppression levels are increased by 13 (UI 11 to 18), 13 (9 to 6) and 12 (8 to 17) percentage points above 2015 levels (increasing from 49% in 2015 to 61%, 60%, and 60%) after 2, 5 and 10 years respectively (Figure 2a; note that the median increase does not always exactly match the difference between the median initial and final levels). Higher suppression levels are needed to achieve a 50% incidence rate reduction (compared to the base‐case scenario) after 2, 5 and 10 years: viral suppression must be increased by 30 (24 to 39), 27 (22 to 35) and 25 (20 to 32) percentage points above 2015 levels respectively (Figure 2a).

The increase in viral suppression needed to reach a 50% incidence rate reduction target after five years is similar across the different demography and diagnosis fitting assumptions (Figure 2b). However, smaller increases in viral suppression are needed under the NHBS ART coverage data fitting assumption than under the DH continuum data assumption. Smaller increases in viral suppression are required to produce the same incidence reduction for higher 2015 viral suppression levels (Figure S6). Subsequent results are pooled across the eight groups of model fits.

#### 3.3.2 Question 2: What care continuum improvements are needed to meet HIV incidence reduction targets?

**Figure 3:**
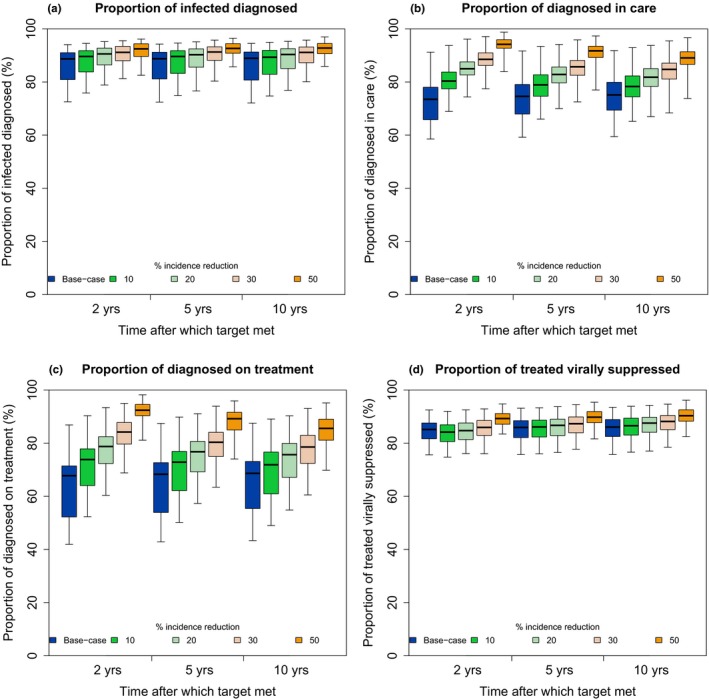
Care continuum indicators in the base‐case scenario (blue) and when incidence reduction targets (compared with the base‐case scenario at the same time point) of 10, 20 30 or 50% (green‐orange) are met after 2, 5 or 10 years, in analyses where all continuum parameters are varied simultaneously These are the continuum indicators for the same results shown in Figure 2a. **(a)** Percentage of all HIV‐positive men who have sex with men (MSM) diagnosed, **(b)** percentage of diagnosed MSM in care, **(c)** percentage of diagnosed MSM on antiretroviral therapy (ART), **(d)** percentage of MSM on ART who are virally suppressed. Box and whiskers are calculated from mean values across all fits (i.e. combining the eight groups of model fits). The thick horizontal line, box and whiskers show the median, 25th to 75th percentiles, and minimum/maximum values respectively.

Due to high 2015 levels of diagnosis and viral suppression amongst those treated, only small improvements in these indicators can be achieved, and so only small increases in these indicators were necessary (along with increases in other indicators) for incidence reduction targets to be met (Figure 3a,d). More substantial increases in care and ART indicators were needed to meet the most ambitious target of 50% incidence reduction – while improving all continuum parameters simultaneously, to meet this target after five years the percentage of diagnosed in care reaches 89%, and diagnosed on ART 86%, from 72% and 66% in 2015 (Figure 3b,c).

Improvements to parameters at every stage of the care continuum were strongly associated with reductions in HIV incidence (correlation coefficient |*r*|>0.4). HIV incidence reductions were most strongly associated with reductions in ART dropout, followed by increases in ART initiation, ART adherence and linkage to care (Figure S7).

#### 3.3.3 Question 3: What reduction in HIV incidence will be achieved if US and UNAIDS targets are met in 2020?

For 43% of fits, the model projects the US new diagnoses target (25% relative decline in new HIV diagnoses between 2010 and 2020) will be met in the base‐case scenario, without continuum parameter improvements, since HIV incidence – and diagnoses – are already declining in the base‐case, and this target uses a historical comparator. On average, this target could be met if viral suppression in 2020 increased by 6 (UI 1 to 23) percentage points above 2015 levels, only producing a 5% (0% to 32%) relative HIV incidence rate reduction compared with the base‐case scenario in 2020 (Figure S8). This small reduction in incidence occurs because the comparator is the base‐case scenario, in which incidence is also declining, rather than a historical comparator.

**Figure 4:**
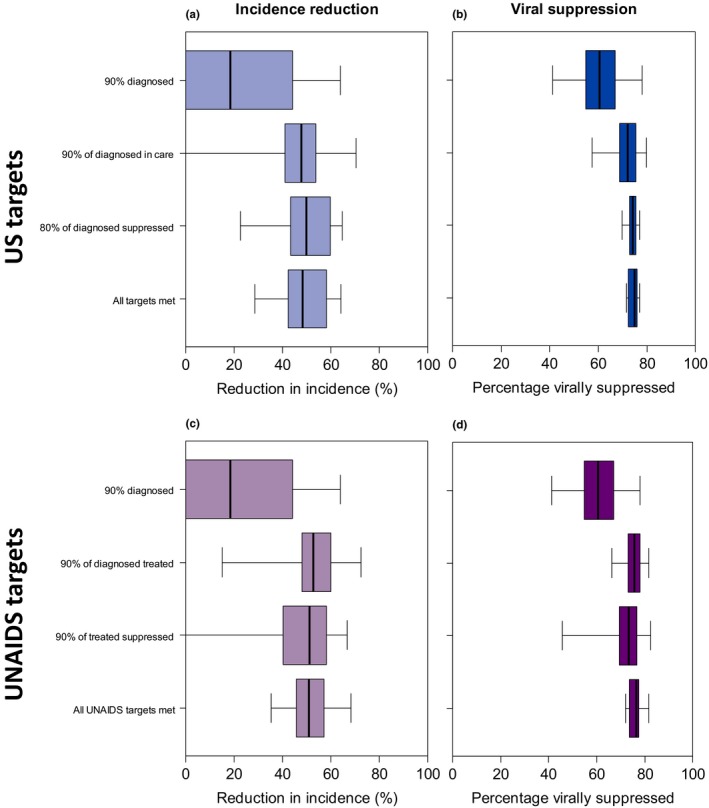
HIV incidence reduction compared with the base‐case scenario in 2020 (a, c) and percentage virally suppressed in 2020 (b, d) when independently or simultaneously meeting the (a, b) US (c, d) UNAIDS continuum targets Results show mean values calculated for each of up to 169 fits (pooled across the eight groups of model fits). The thick horizontal line, box and whiskers show the median, 25th to 75th percentiles, and minimum/maximum values.

Achieving the 90% diagnosis target in 2020 (while improving all care continuum parameters simultaneously) has a limited impact, reducing the HIV incidence rate (compared with base‐case in 2020) by 18% (Figure 4a). However, greater reductions in HIV incidence rates (48% to 50%) are achieved in 2020 by either attaining the US continuum indicator targets of 90% diagnosed in care, 80% of diagnosed virally suppressed, or all three continuum targets simultaneously, giving 72% to 75% of MSM virally suppressed in 2020 (Figure 4a,b).

Meeting the three UNAIDS continuum targets together results in similar HIV incidence reduction (51%) and viral suppression level (76%) as meeting US continuum targets (48% incidence reduction, 75% virally suppressed; Figure 4c,d).

#### 3.3.4 Question 4: What reduction in HIV incidence will be seen if the HPTN 078 trial target effect size is achieved?

If the percentage of diagnosed MSM virally suppressed is 18 percentage points higher in the intervention than the base‐case scenario after two years, the model projects a 35% (UI 21% to 43%) reduction in HIV incidence versus base‐case (data not shown).

#### 3.3.5 Question 5: What is the potential for local HIV elimination?

HIV elimination (incidence <1/1000 person‐years) is never achieved before 2036 in base‐case scenarios, and is unlikely to be achieved by 2036 under any of the incidence reduction, US, or UNAIDS targets investigated, occurring in at most 6% of model runs (Figure S9, Table S3).

## 4 Discussion

Our model results provide important insights for HIV prevention in Baltimore and the US. First, our results suggest HIV incidence is already declining among Baltimore MSM under current rates of HIV testing, linkage and ART initiation. This means the US new diagnoses target (25% relative decline in new HIV diagnoses between 2010 and 2020) could be met with minor continuum improvements, but this is expected to only produce a small (5%) reduction in HIV incidence rate in 2020 compared with base‐case scenarios where current HIV testing, linkage, and ART initiation rates are maintained. Second, large increases in viral suppression – associated with large ART coverage increases – are needed to significantly reduce HIV incidence rates among MSM in Baltimore compared with base‐case scenario projections. To reduce HIV incidence rates by 50% compared to base‐case after five years, viral suppression levels must increase by 27 percentage points above 2015 levels, and 13 percentage points to reduce incidence by 20%. Third, the small HIV incidence reduction projected when meeting the US new diagnoses target contrasts with the 48% reduction in HIV incidence rate expected from meeting US continuum targets, highlighting poor alignment between the new diagnoses and continuum targets. Fourth, local elimination of HIV (incidence below 0.1%/year) among Baltimore MSM is unlikely to occur in the next 20 years, even if US or UNAIDS targets are reached, suggesting additional primary prevention efforts (e.g. pre‐exposure prophylaxis (PrEP) or a vaccine) are needed to achieve elimination. Fifth, our results emphasize the importance of achieving low rates of ART dropout, and high ART initiation rates and adherence levels to achieve substantial HIV incidence reductions.

### 4.1 Limitations

Where possible, we used data from Baltimore. However, some behavioural parameters were sourced from other US locations. Therefore, we may not have fully captured local care continuum progression. Our model did not fully capture the decline in prevalence among older white MSM in the 2014 NHBS. This lower prevalence could partly have resulted from different venues being sampled in the 2014 NHBS than at earlier surveys. Overestimating HIV prevalence among white MSM could mean we underestimated base‐case scenario HIV incidence declines, although since our model captured prevalence trends among black MSM – who account for most HIV‐positive MSM in Baltimore – very well, this is unlikely to greatly impact our results. Due to limited data, we excluded MSM of Hispanic ethnicity, who are a small proportion (<5%) of MSM in Baltimore but have low viral suppression (43% in 2015 5), due to low levels of engagement in care and suboptimal suppression among those in care. New diagnoses among Hispanic MSM in Maryland have declined in recent years proportionately to overall MSM diagnoses 27, suggesting no significant local incidence differences among Hispanic MSM which might affect our results. NHBS data are collected only from MSM recruited from MSM‐identified venues, and may not reflect the demographic, behavioural and HIV‐related characteristics of the broader Baltimore MSM population (although we showed that our results were not sensitive to NHBS demography and HIV testing data – Figure 2b). In fits to DH continuum data, our model underestimated levels of viral suppression over the period 2014 to 2017; however, as we also fitted the model to ART coverage data, our results cover situations where viral suppression is similar to DH 2014 to 2017 estimates. In intervention scenarios we assumed instantaneous care continuum parameter improvements, therefore our estimates likely overestimate achievable impact, particularly in the short‐term (1 to 2 years). Transmission via injection drug use was not included in our model. In DH surveillance, only 14% of diagnosed PLHIV with MSM exposure also have injecting drug use exposure 5, suggesting little overlap between the MSM and injection drug use epidemics.

Few studies have assessed the potential impact upon HIV incidence of meeting UNAIDS 90‐90‐90 targets in the US. Risk equation modelling projected that if the US reached the UNAIDS 90‐90‐90 targets by 2020 at the national level, HIV incidence could be reduced by 50% 28, aligning closely with our 51% incidence reduction projection for meeting UNAIDS targets among Baltimore MSM. Another modelling study projected a slightly smaller 40% reduction in HIV incidence 29. A recent modelling study suggested that meeting the US target to have 90% of diagnosed PLHIV in care by 2020 would reduce US national HIV incidence (vs. base‐case) by around 56% 30, higher than our projected 48% reduction – this is likely due to lower baseline levels of PLHIV in care in their study. In agreement with our findings, they found a much smaller impact of reaching the 90% diagnosed target 30. Another modelling study suggested slightly more modest national impact (46% incidence decline from 2013 to 2020) when US 2020 targets are met 31.

Baltimore is currently expanding access to PrEP for HIV at‐risk and uninfected adults, including MSM. PrEP was not included in this analysis as reported use in the 2014 Baltimore MSM NHBS was very low, and more recent city‐level coverage data are not yet available. State‐level estimates of PrEP usage 27 suggest around 2% of uninfected MSM in Maryland are using PrEP. This level of use would minimally affect our results; however, substantial scale‐up of PrEP in the near future would mean that smaller increases in viral suppression would be needed to meet HIV incidence reduction targets. Data from other communities with high MSM HIV burdens suggest that improvements in the care continuum for HIV‐positive MSM and significant levels of PrEP uptake can have synergistic impacts on HIV incidence 32, 33.

Our analysis has highlighted incompatibilities between different data sources, including current levels of viral suppression among MSM in Baltimore. Estimates of ART coverage obtained from antiretroviral detection in plasma in the Baltimore MSM NHBS sub‐study suggest higher levels of viral suppression (59% for model fits, 2015) than DH estimates of viral suppression among diagnosed MSM (data estimate 46%, 2015). As NHBS participants may not be representative of the wider MSM population the sub‐study could have overestimated true ART coverage. DH statistics may underestimate viral suppression levels, as viral load measurements were only available for 54% of diagnosed Baltimore MSM in 2015. Our results fully account for the resulting uncertainty, with uncertainty ranges including pessimistic and optimistic values, but as this uncertainty influenced our results, these differences warrant further investigation.

The model projects that if the HPTN 078 trial achieves its target effect size – an 18 percentage point difference in viral suppression levels between trial arms after two years – this would achieve a 35% reduction in incidence compared with the base‐case scenario. Greater differences – approximately 30 percentage points – are needed to achieve 50% incidence reduction.

Future modelling studies should assess the efforts needed and resulting impact of meeting these targets in other US settings. They should also consider in what ways prioritizing and investing in interventions to groups most affected by HIV transmission could increase efficiency in these settings, and whether advances in primary prevention, including oral PrEP, can further accelerate incidence declines and achieve elimination.

## 5 Conclusions

Our model suggests HIV incidence is already declining among MSM in Baltimore, but to achieve more rapid HIV incidence reductions, more intense efforts are needed to strengthen multiple points along the care continuum, particularly improving linkage to care, ART retention and long‐term adherence. This will require substantial investment and attention to the multi‐layered social and structural influences affecting Baltimore MSM, especially black MSM 34. Meeting US and UNAIDS targets is likely to reduce HIV incidence rates by around 50% in 2020, but will require large increases in ART coverage, and additional primary prevention efforts will be needed to achieve HIV elimination in the near future.

## Competing interests

The authors declare that they have no competing interests.

## Authors’ contributions

Marie‐Claude Boily, Dobromir Dimitrov, Kate M Mitchell, Gabriela Paz‐Bailey, Danielle German, Colin Flynn, James P. Hughes, Deborah Donnell, Adeola Adeyeye, Robert H Remien and Chris Beyrer contributed to study conceptualization. Brooke Hoots and Gabriela Paz‐Bailey curated the NHBS data. Kate M Mitchell conducted the modelling and Brooke Hoots conducted the main NHBS data analysis. Marie‐Claude Boily, Deborah Donnell, Chris Beyrer and Robert H Remien acquired funding. Kate M Mitchell, Danielle German, Colin Flynn, Jason E. Farley, Marcy Gelman, Gabriela Paz‐Bailey contributed to other data analyses. Kate M Mitchell, Marie‐Claude Boily and Dobromir Dimitrov designed the modelling methodology. Kate M Mitchell developed the model code. Marie‐Claude Boily oversaw the study. Kate M Mitchell and Marie‐Claude Boily wrote the initial draft of the manuscript. Kate M Mitchell, Brooke Hoots, Dobromir Dimitrov, Danielle German, Colin Flynn, Jason E. Farley, Marcy Gelman, James P. Hughes, Deborah Donnell, Adeola Adeyeye, Robert H Remien, Chris Beyrer, Gabriela Paz‐Bailey and Marie‐Claude Boily reviewed and edited the manuscript. All authors have read and approved the final manuscript.

## Supporting information


**Figure S1:** Age groups, race groups, movement and mixing in the model.
**Figure S2:** HIV disease progression, by HIV states and set‐point viral load, for those not on antiretroviral therapy (ART), and for those on ART but not adherent.
**Figure S3:** Different stages of HIV care and transitions between them.
**Figure S4:** Illustration of calculations of targets and outcomes.
**Figure S5:** Outcomes for model fits.
**Figure S6:** Required increase in viral suppression in 2020 above initial 2015 level needed to meet a 50% incidence reduction compared with the base‐case scenario in 2020, for all runs across all fits, against initial level of viral suppression in 2015.
**Figure S7:** Correlations between the increase in care continuum parameters above 2015 levels (*y*‐axis) and the relative reduction in HIV incidence after five years, for all runs.
**Figure S8:** New HIV diagnoses over time under the base‐case scenario and when different US targets are met in 2020.
**Figure S9:** HIV incidence (new cases per 100 uninfected men who have sex with men per year) over time under the base‐case scenario and when different targets are met.
**Table S1:** Parameters used in the HIV transmission model, with source and justification.
**Table S2:** Data fitted to, with fitting bounds, source and justification.
**Table S3:** Probability of reaching HIV elimination within 20 years when meeting different (a) trial incidence reduction targets after different time periods; (b) US continuum targets in 2020; (c) UNAIDS targets in 2020.
**Appendix S1:** Methods.
