# Supplementary material for: Improvements in the HIV care continuum needed to meaningfully reduce HIV incidence among men who have sex with men in Baltimore, US: a modelling study for HPTN 078
Source: J Int AIDS Soc. 2019 Mar 14;22(3):e25246. doi: 10.1002/jia2.25246 (PMC6416473; doi:10.1002/jia2.25246)
Supplement: Supplementary file 1 — Figure S1: Age groups, race groups, movement and mixing in the model. Figure S2: HIV disease progression, by HIV states and set‐point viral load, for those not on antiretroviral therapy (ART), and for those on ART but not adherent. Figure S3: Different stages of HIV care and transitions between them. Figure S4: Illustration of calculations of targets and outcomes. Figure S5: Outcomes for model fits. Figure S6: Required increase in viral suppression in 2020 above initial 2015 level needed to meet a 50% incidence reduction compared with the base‐case scenario in 2020, for all runs across all fits, against initial level of viral suppression in 2015. Figure S7: Correlations between the increase in care continuum parameters above 2015 levels (y‐axis) and the relative reduction in HIV incidence after five years, for all runs. Figure S8: New HIV diagnoses over time under the base‐case scenario and when different US targets are met in 2020. Figure S9: HIV incidence (new cases per 100 uninfected men who have sex with men per year) over time under the base‐case scenario and when different targets are met. Table S1: Parameters used in the HIV transmission model, with source and justification. Table S2: Data fitted to, with fitting bounds, source and justification. Table S3: Probability of reaching HIV elimination within 20 years when meeting different (a) trial incidence reduction targets after different time periods; (b) US continuum targets in 2020; (c) UNAIDS targets in 2020. Appendix S1: Methods. [file JIA2-22-e25246-s001.pdf]

Supporting Information

Improvements in the HIV care continuum needed to meaningfully reduce HIV incidence among men who have sex with men in Baltimore, US: a modelling study for HPTN 078

Kate M. Mitchell et al.

METHODS

Model structure

In the model equations and schematics, uninfected MSM are denoted by $X^{z}_{v,w}$, those with acute HIV infection by $A^{z}_{v,w}$ and chronic HIV infection by $Y^{x,y,z}_{v,w}$. Subscripts refer to the following states: v is age group (0 = 18-24 years old; 1 = >24 years old), w is race (0 = black; 1 = white). The younger age group had a lower age limit of 18 to match the minimum age of MSM included in NHBS surveys, which supplied the behavioural parameters and HIV prevalence estimates used in this analysis. Superscripts refer to the following states: x is CD4 count (current CD4 count for those not taking or not adherent to ART, CD4 count at ART initiation for those taking and adherent to ART; 0 = acute, 1 = CD4>500, 2 = CD4 350-500, 3 = CD4 200-350, 4 = CD4 <200 cells/µl), y is set-point viral load (SPVL; 0 = acute, 1 = Log_{10} SPVL<4.0, 2 = Log_{10} SPVL 4.0-4.5, 3 = Log_{10} SPVL 4.5-5.0, 4 = Log_{10} SPVL >5.0), z is care state (0 = never testing, 1 = testing but not diagnosed, 2 = diagnosed not linked to care, 3 = linked into HIV care, 4 = on ART, adherent and partially suppressed, 5 = in first year on ART, adherent and fully suppressed, 6 = 2nd year on ART adherent and fully suppressed, 7 = 3rd and subsequent years on ART adherent and fully suppressed, 8 = on ART but non-adherent and not suppressed, 9 = stopped taking ART (due to dropout or failure)). For those uninfected with HIV, the only possible care states are z=0 or 1. Those with acute infection may be in one of care states z=0-4; after achieving full viral suppression on ART they are assumed to no longer be in the acute stage.

Fig S1 shows the age and race groups, with movement and sexual mixing between them. Of individuals entering the sexually active Baltimore MSM population, a proportion $m_{v,w}$ are assumed to be in each combination of age and race group ($m_{v,w}$ is calculated from m_{black}, the proportion of incoming MSM who are black, and $m_{young,w}$, the proportion of incoming MSM of each race who are aged 18-24 years old. Those in the 18-24 year old group move

into the older age group at an annual rate π_w per year, corresponding to an average of $1/\ \pi_w$ years that sexually active MSM in race group w spend in the 18-24 year old age group.

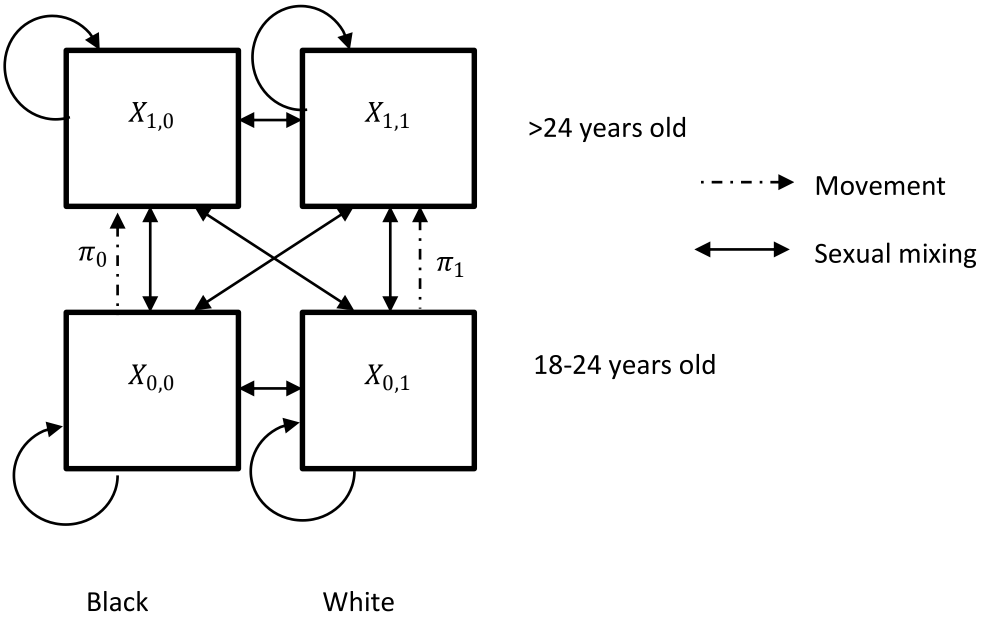

Fig S1: Age groups, race groups, movement and mixing in the model

Fig S2 shows the transitions between different stages of HIV infection for those not currently taking ART, by current HIV stage and SPVL. These transitions are the same for all age, race and care states (apart from those on ART and adherent), with the following exceptions: infection rates (λ) and background death rate (μ) differ by age and race.

Susceptible individuals (X^z) become infected with HIV at a rate $\lambda_{v,w}$ and move into the acutely infected compartment (A^z). After a period ($1/\gamma_a$ years) in the acute stage, individuals move into one of 16 compartments ($Y^{x,y,z}$), defined by their SPVL and initial CD4 count after acute infection. A proportion (θ_y) of those leaving the acute stage move into SPVL stratum y. For each SPVL stratum, a proportion $f_{x,y}$ of those entering SPVL stratum y are initially in CD4 compartment $Y^{x,y,z}$. Within each SPVL stratum, HIV-positive people pass sequentially through progressively lower CD4 count categories. The rate of moving from one CD4 compartment to the next is given by $\gamma_{x,y}$.

There is a constant background per-capita rate of non-HIV related death ($\mu_{v,w}$) from every compartment (susceptibles and all infected compartments), and an additional rate of HIV–

related death from each infected compartment ($\alpha_{x,y,z}$), which varies by SPVL and current CD4 count, but takes the same value for all those off ART (z = 0,1,2,3,8,9).

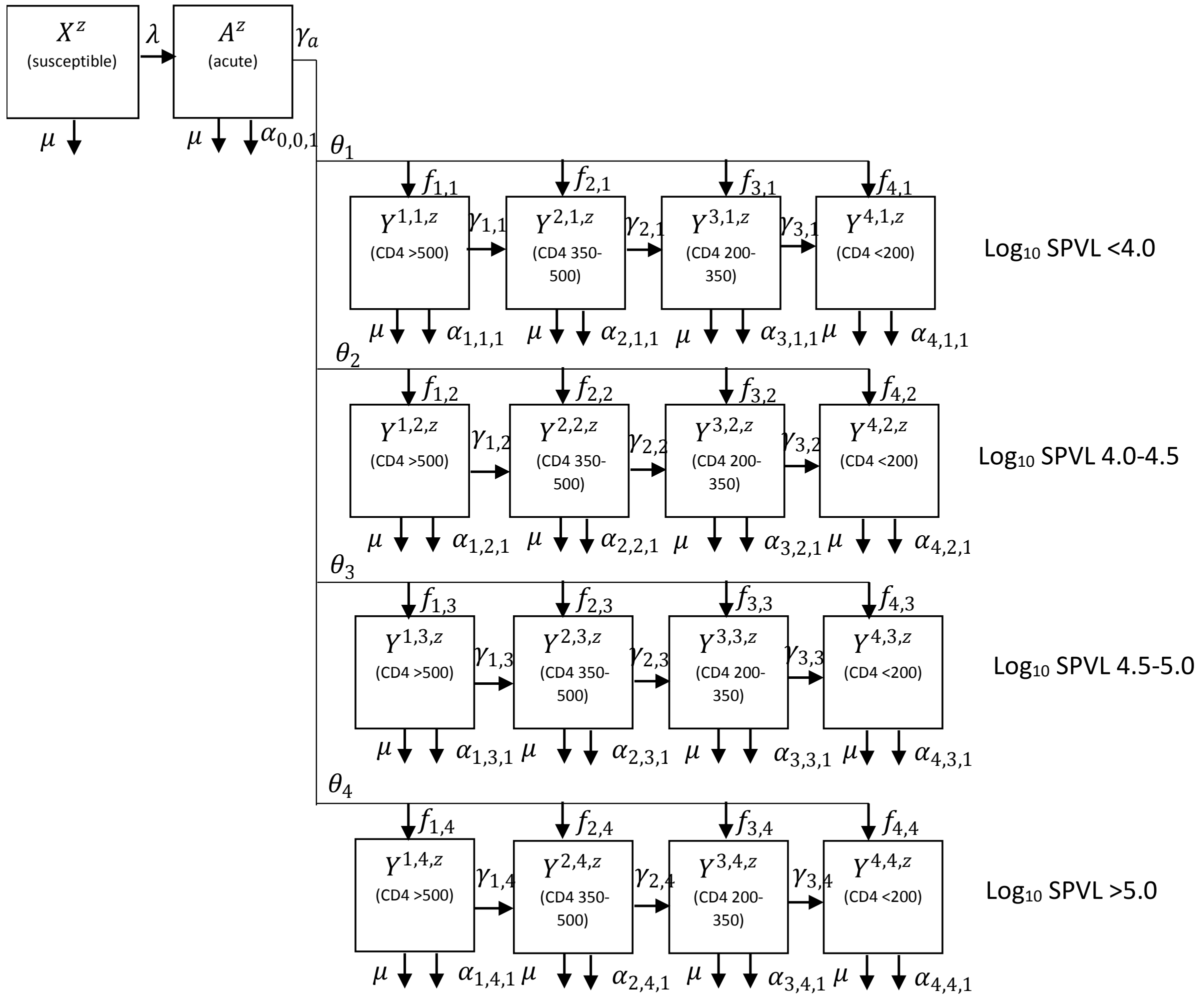

Fig S2. HIV disease progression, by HIV states and SPVL, for those not on ART, and for those on ART but not adherent. Superscripts on states and HIV-related death rates are x,y,z (x = CD4 category; y = set-point viral load category; z = care state); subscripts for age and race are omitted for clarity.

Transitions between the different stages of care are shown in Fig S3. New men join the sexually active MSM population (through ageing into the population, sexual debut or immigration), at a rate Γ and are assumed to all be uninfected with HIV initially. A proportion p of new entrants are assumed to never routinely test for HIV and do not seek treatment until they become symptomatic (develop AIDS-defining illness); they enter the compartment for never-testing susceptibles (X^0). The remainder of new entrants enter compartment X^1, who are susceptibles who may undergo HIV testing. Susceptibles in either state may become infected at a rate λ. Those never testing who become infected enter the

infected compartments of never testers ($A^0/Y^{x,y,0}$, for acute/chronic infection, respectively). Those who may test enter the infected compartments of those undiagnosed but who may undergo HIV testing ($A^1/Y^{x,y,1}$). These individuals undergo HIV testing at a per-capita rate $\tau_{v,w}$ (which varies with age and race); a proportion q of those testing are rapidly linked into care and move into the 'in care' compartments $A^3/Y^{x,y,3}$, the remainder (1-q) move into the 'diagnosed not linked into care' compartment ($A^2/Y^{x,y,2}$). Those who are diagnosed but not in care can be linked into care, moving into compartments $A^3/Y^{x,y,3}$ at a rate $\epsilon_{v,w}$, and those linked into care may drop out from pre-ART care and go into the 'diagnosed not linked into care' compartment ($A^2/Y^{x,y,2}$) at a rate $\omega_w\phi_4$ (ϕ_4 is the rate of dropout from ART in the first year of treatment, $\omega_w$is the race-specific ratio of dropout from care relative to rate of dropout from ART). Those linked into care may begin ART, at a rate related to their CD4 count, ξ_x, with a proportion (χ) who are adherent to their treatment moving into the first ART compartment, $A^4/ Y^{x,y,4}$, and those who are non-adherent (1-χ) moving into compartment , $Y^{x,y,8}$. People at any other stage of the care continuum may also begin ART due to becoming symptomatic and seeking medical care, at a rate $\psi_{x,z}$, which is based upon CD4-count specific rates of incidence of AIDS-defining illness, and whether or not they have previously taken ART, and also move into the first ART compartment if they are adherent (proportion χ), or the "on ART but not adherent" compartment ($Y^{x,y,8}$) if they are not adherent. Those in the non-adherent ART compartment are assumed to be fully infectious and have no survival benefit from ART, and progress in the same way as those not on ART.

People in the first ART compartment, $A^4/ Y^{x,y,4}$, are assumed to be partially virally suppressed, and they leave this compartment at a rate σ_y, where 1/σ_y is the average duration from ART initiation to achieving viral suppression. σ_y varies by SPVL, but not by initial CD4 count [1]. They move into the first fully virally suppressed compartment ($Y^{x,y,5}$), where they stay for the remainder of their first year on ART, and move into the next ART compartment (2nd year;$Y^{x,y,6}$) at a rate η_y, where 1/η_y (the average duration spent in the first year compartment) is estimated as 1- 1/σ_y. People move from the 2nd year on ART compartment ($Y^{x,y,6}$) into the >2 years on ART compartment ($Y^{x,y,7}$) at a rate 1/year. The final fully suppressed compartment ($Y^{x,y,7}$) contains those who have remained on ART for more than 2 years and are still virally suppressed. For those on ART, the additional rate of HIV–related death from each of these compartments ($\alpha_{x,y,z}$) varies by CD4 count at ART initiation and duration on ART.

Those in any of the ART compartments may drop out of treatment at a rate ϕ_z, which varies with time since initiation of ART. Dropouts from ART go initially into the dropout compartments, $Y^{x,y,9}$, where they progress through different CD4 compartments in the same way as those never on ART. Those dropping out of the adherent ART compartments ($A^4, Y^{x,y,4} - Y^{x,y,7}$), move into the same CD4 compartment as the one they were in when they started ART , those dropping out of the non-adherent ART compartment ($Y^{x,y,8}$) retain the CD4 count they had at the point of dropout. People remain in the same SPVL category after dropping out of ART. ART dropouts may re-initiate treatment due to developing AIDS symptoms and seeking medical care, at a rate $\psi_{x,9}$, or may re-enrol in HIV care, at a rate ζ. Those re-entering care are not distinguished from those entering care for the first time. Likewise, those re-initiating treatment progress in the same way as those beginning ART for the first time, and are not distinguished from them.

We do not explicitly model individuals on ART gaining and losing viral suppression over time, due to a lack of data, but we do capture overall levels of viral suppression as well as dynamic (re-)entry and dropout from care and treatment.

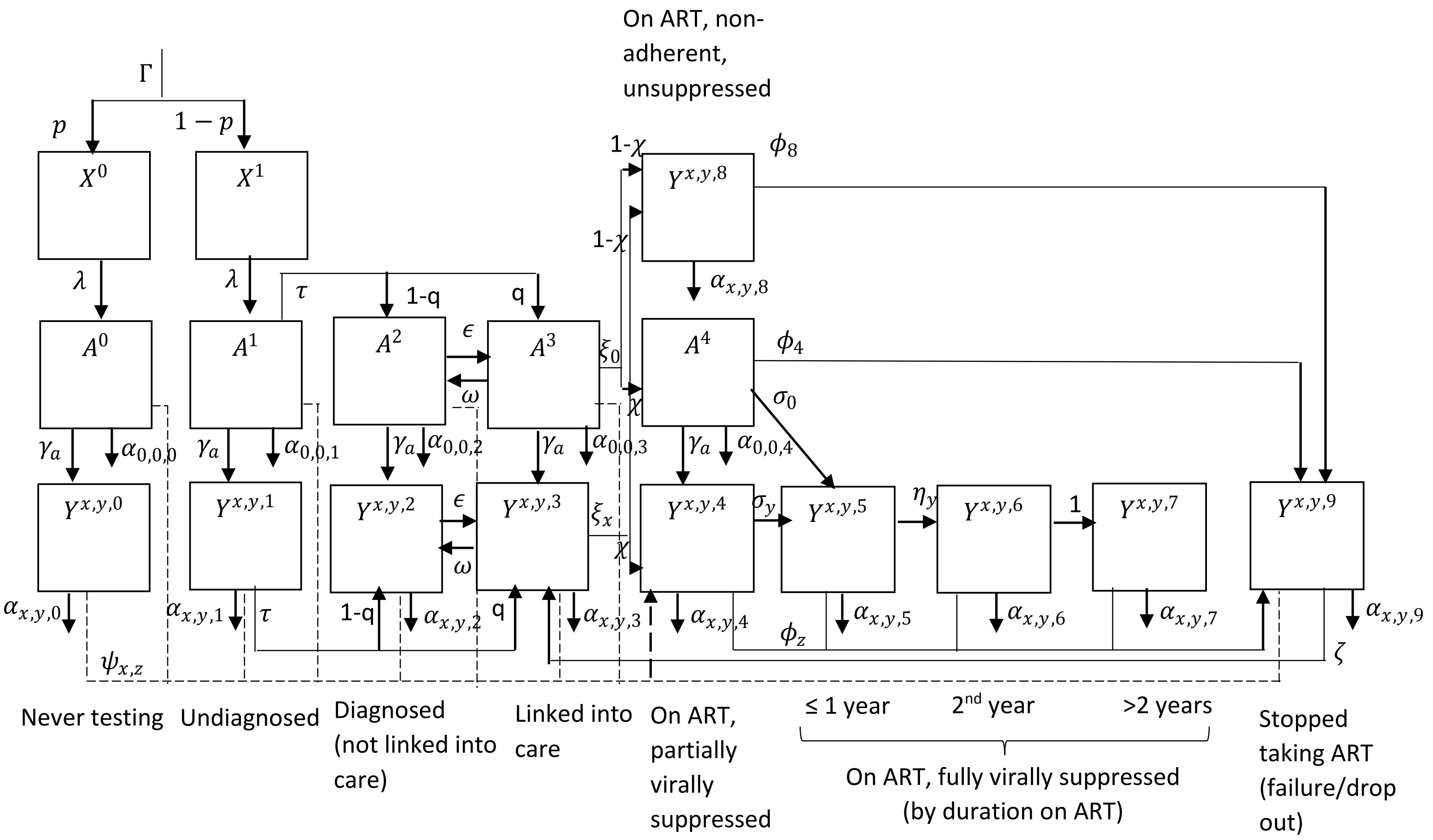

Fig S3: Different stages of HIV care and transitions between them

Model equations

MSM who never get tested for HIV:

$$\frac{d}{dt}\left(X_{v,w}^{0}\right) = \Gamma p m_{v,w} + v\pi_w X_{1-v,w}^{0} - X_{v,w}^{0}\left(\lambda_{v,w,1} + \mu_{v,w} + (1-v)\pi_w\right)$$

$$\frac{d}{dt}\left(A_{v,w}^{0}\right) = \lambda_{v,w,1} X_{v,w}^{0} + v\pi_w A_{1-v,w}^{0} - A_{v,w}^{0}\left(\gamma_a + \mu_{v,w} + \alpha_{0,0,0} + (1-v)\pi_w + \psi_{0,0}\right)$$

$$\frac{d}{dt}\left(Y_{v,w}^{1,y,0}\right) = \gamma_a \theta_y f_{1,y} A_{v,w}^{0} + v\pi_w Y_{1-v,w}^{1,y,0} - Y_{v,w}^{1,y,0}\left(\gamma_{1,y} + \mu_{v,w} + \alpha_{1,y,0} + (1-v)\pi_w + \psi_{1,0}\right)$$

$$\frac{d}{dt}\left(Y_{v,w}^{x,y,0}\right) = \gamma_a \theta_y f_{x,y} A_{v,w}^{0} + \gamma_{x-1,y} Y_{v,w}^{x-1,y,0} + v\pi_w Y_{1-v,w}^{x,y,0} - Y_{v,w}^{x,y,0}\left(\gamma_{x,y} + \mu_{v,w} + \alpha_{x,y,0} + (1-v)\pi_w + \psi_{x,0}\right); x \in \{2,3\}$$

$$\frac{d}{dt}\left(Y_{v,w}^{4,y,0}\right) = \gamma_a \theta_y f_{4,y} A_{v,w}^{0} + \gamma_{3,y} Y_{v,w}^{3,y,0} + v\pi_w Y_{1-v,w}^{4,y,0} - Y_{v,w}^{4,y,0}\left(\mu_{v,w} + \alpha_{4,y,0} + (1-v)\pi_w + \psi_{4,0}\right)$$

MSM who may get tested, not diagnosed:

$$\frac{d}{dt}\left(X_{v,w}^{1}\right) = \Gamma(1-p) m_{v,w} + v\pi_w X_{1-v,w}^{1} - X_{v,w}^{1}\left(\lambda_{v,w,1} + \mu_{v,w} + (1-v)\pi_w\right)$$

$$\frac{d}{dt}\left(A_{v,w}^{1}\right) = \lambda_{v,w,1} X_{v,w}^{1} + v\pi_w A_{1-v,w}^{1} - A_{v,w}^{1}\left(\gamma_a + \mu_{v,w} + \alpha_{0,0,1} + (1-v)\pi_w + \psi_{0,1} + \tau_{v,w}\right)$$

$$\frac{d}{dt}\left(Y_{v,w}^{1,y,1}\right) = \gamma_a \theta_y f_{1,y} A_{v,w}^{1} + v\pi_w Y_{1-v,w}^{1,y,1} - Y_{v,w}^{1,y,1}\left(\gamma_{1,y} + \mu_{v,w} + \alpha_{1,y,1} + (1-v)\pi_w + \psi_{1,1} + \tau_{v,w}\right)$$

$$\frac{d}{dt}\left(Y_{v,w}^{x,y,1}\right) = \gamma_a \theta_y f_{x,y} A_{v,w}^1 + \gamma_{x-1,y} Y_{v,w}^{x-1,y,1} + v\pi_w Y_{1-v,w}^{x,y,1} - Y_{v,w}^{x,y,1}\left(\gamma_{x,y} + \mu_{v,w} + \alpha_{x,y,1} + (1-v)\pi_w + \psi_{x,1} + \tau_{v,w}\right); x \in \{2,3\}$$

$$\frac{d}{dt}\left(Y_{v,w}^{4,y,1}\right) = \gamma_a \theta_y f_{4,y} A_{v,w}^1 + \gamma_{3,y} Y_{v,w}^{3,y,1} + v\pi_w Y_{1-v,w}^{4,y,1} - Y_{v,w}^{4,y,1}\left(\mu_{v,w} + \alpha_{4,y,1} + (1-v)\pi_w + \psi_{4,1} + \tau_{v,w}\right)$$

MSM diagnosed but not in care:

$$\frac{d}{dt}\left(A_{v,w}^2\right) = v\pi_w A_{1-v,w}^2 + (1-q)\tau_{v,w} A_{v,w}^1 + \omega_w \phi_4 A_{v,w}^3 - A_{v,w}^2\left(\gamma_a + \mu_{v,w} + \alpha_{0,0,2} + (1-v)\pi_w + \psi_{0,2} + \epsilon_w\right)$$

$$\frac{d}{dt}\left(Y_{v,w}^{1,y,2}\right) = \gamma_a \theta_y f_{1,y} A_{v,w}^2 + v\pi_w Y_{1-v,w}^{1,y,2} + (1-q)\tau_{v,w} Y_{v,w}^{1,y,1} + \omega_w \phi_4 Y_{v,w}^{1,y,3} - Y_{v,w}^{1,y,2}\left(\gamma_{1,y} + \mu_{v,w} + \alpha_{1,y,2} + (1-v)\pi_w + \psi_{1,2} + \epsilon_w\right)$$

$$\frac{d}{dt}\left(Y_{v,w}^{x,y,2}\right) = \gamma_a \theta_y f_{x,y} A_{v,w}^2 + \gamma_{x-1,y} Y_{v,w}^{x-1,y,2} + v\pi_w Y_{1-v,w}^{x,y,2} + (1-q)\tau_{v,w} Y_{v,w}^{x,y,1} + \omega_w \phi_4 Y_{v,w}^{x,y,3} - Y_{v,w}^{x,y,2}\left(\gamma_{x,y} + \mu_{v,w} + \alpha_{x,y,2} + (1-v)\pi_w + \psi_{x,2} + \epsilon_w\right); x \in \{2,3\}$$

$$\frac{d}{dt}\left(Y_{v,w}^{4,y,2}\right) = \gamma_a \theta_y f_{4,y} A_{v,w}^2 + \gamma_{3,y} Y_{v,w}^{3,y,2} + v\pi_w Y_{1-v,w}^{4,y,2} + (1-q)\tau_{v,w} Y_{v,w}^{4,y,1} + \omega_w \phi_4 Y_{v,w}^{4,y,3} - Y_{v,w}^{4,y,2}\left(\mu_{v,w} + \alpha_{4,y,2} + (1-v)\pi_w + \psi_{4,2} + \epsilon_w\right)$$

MSM in care:

$$\frac{d}{dt}\left(A_{v,w}^{3}\right) = v\pi_w A_{1-v,w}^{3} + q\tau_{v,w}A_{v,w}^{1} + \epsilon_w A_{v,w}^{2} - A_{v,w}^{3}\left(\gamma_a + \mu_{v,w} + \alpha_{0,0,3} + (1-v)\pi_w + \psi_{0,3} + \omega_w\phi_4 + \xi_0\right)$$

$$\frac{d}{dt}\left(Y_{v,w}^{1,y,3}\right) = \gamma_a\theta_y f_{1,y}A_{v,w}^{3} + v\pi_w Y_{1-v,w}^{1,y,3} + q\tau_{v,w}Y_{v,w}^{1,y,1} + \epsilon_w Y_{v,w}^{1,y,2} + \zeta Y_{v,w}^{1,y,8} - Y_{v,w}^{1,y,3}\left(\gamma_{1,y} + \mu_{v,w} + \alpha_{1,y,3} + (1-v)\pi_w + \psi_{1,3} + \omega_w\phi_4 + \xi_1\right)$$

$$\frac{d}{dt}\left(Y_{v,w}^{x,y,3}\right) = \gamma_a\theta_y f_{x,y}A_{v,w}^{3} + \gamma_{x-1,y}Y_{v,w}^{x-1,y,3} + v\pi_w Y_{1-v,w}^{x,y,3} + q\tau_{v,w}Y_{v,w}^{x,y,1} + \epsilon_w Y_{v,w}^{x,y,2} + \zeta Y_{v,w}^{x,y,8} - Y_{v,w}^{x,y,3}\left(\gamma_{x,y} + \mu_{v,w} + \alpha_{x,y,3} + (1-v)\pi_w + \psi_{x,3} + \omega_w\phi_4 + \xi_x\right); x \in \{2,3\}$$

$$\frac{d}{dt}\left(Y_{v,w}^{4,y,3}\right) = \gamma_a\theta_y f_{4,y}A_{v,w}^{3} + \gamma_{3,y}Y_{v,w}^{3,y,3} + v\pi_w Y_{1-v,w}^{4,y,3} + q\tau_{v,w}Y_{v,w}^{4,y,1} + \epsilon_w Y_{v,w}^{4,y,2} + \zeta Y_{v,w}^{4,y,8} - Y_{v,w}^{4,y,3}\left(\mu_{v,w} + \alpha_{4,y,3} + (1-v)\pi_w + \psi_{4,3} + \omega_w\phi_4 + \xi_4\right)$$

MSM on ART and adherent:

$$\frac{d}{dt}\left(A_{v,w}^{4}\right) = v\pi_w A_{1-v,w}^{4} + \chi\xi_0 A_{v,w}^{3} + \sum_{Z=0}^{Z=3}\chi\psi_{0,Z}A_{v,w}^{Z} - A_{v,w}^{4}\left(\gamma_a + \mu_{v,w} + \alpha_{0,0,4} + (1-v)\pi_w + \sigma_0 + \phi_4\right)$$

$$\frac{d}{dt}\left(Y_{v,w}^{x,y,4}\right) = \gamma_a \theta_y f_{x,y} A_{v,w}^{4} + v\pi_w Y_{1-v,w}^{x,y,4} + \chi \xi_x Y_{v,w}^{x,y,3} + \sum_{Z=0}^{Z=3} \chi \psi_{x,Z} Y_{v,w}^{x,y,Z} + \chi \psi_{x,8} Y_{v,w}^{x,y,8} - Y_{v,w}^{x,y,4}\left(\mu_{v,w} + \alpha_{x,y,4} + (1-v)\pi_w + \sigma_y + \phi_4\right)$$

$$\frac{d}{dt}\left(Y_{v,w}^{x,y,5}\right) = v\pi_w Y_{1-v,w}^{x,y,5} + \sigma_0 \theta_y f_{x,y} A_{v,w}^{4} + \sigma_y Y_{v,w}^{x,y,4} - Y_{v,w}^{x,y,5}\left(\mu_{v,w} + \alpha_{x,y,5} + (1-v)\pi_w + \eta_y + \phi_5\right)$$

$$\frac{d}{dt}\left(Y_{v,w}^{x,y,6}\right) = v\pi_w Y_{1-v,w}^{x,y,6} + \eta_y Y_{v,w}^{x,y,5} - Y_{v,w}^{x,y,6}\left(\mu_{v,w} + \alpha_{x,y,6} + (1-v)\pi_w + 1 + \phi_6\right)$$

$$\frac{d}{dt}\left(Y_{v,w}^{x,y,7}\right) = v\pi_w Y_{1-v,w}^{x,y,7} + Y_{v,w}^{x,y,6} - Y_{v,w}^{x,y,7}\left(\mu_{v,w} + \alpha_{x,y,7} + (1-v)\pi_w + \phi_7\right)$$

MSM on ART but non-adherent:

$$\frac{d}{dt}\left(Y_{v,w}^{1,y,8}\right) = (1-\chi)\xi_0\theta_y f_{1,y} A_{v,w}^{3} + (1-\chi)\xi_1 Y_{v,w}^{1,y,3} + \sum_{Z=0}^{Z=3}(1-\chi)\psi_{0,Z}\theta_y f_{1,y} A_{v,w}^{Z} + \sum_{Z=0}^{Z=3}(1-\chi)\psi_{1,Z} Y_{v,w}^{1,y,Z} + (1-\chi)\psi_{x,9} Y_{v,w}^{1,y,9} + v\pi_w Y_{1-v,w}^{1,y,8} - Y_{v,w}^{1,y,8}\left(\gamma_{1,y} + \mu_{v,w} + \alpha_{1,y,8} + (1-v)\pi_w + \phi_8\right)$$

$$\frac{d}{dt}\left(Y_{v,w}^{x,y,8}\right) = (1-\chi)\xi_0\theta_y f_{x,y} A_{v,w}^{3} + (1-\chi)\xi_1 Y_{v,w}^{x,y,3} + \sum_{Z=0}^{Z=3}(1-\chi)\psi_{0,Z}\theta_y f_{x,y} A_{v,w}^{Z} + \sum_{Z=0}^{Z=3}(1-\chi)\psi_{x,Z} Y_{v,w}^{x,y,Z} + (1-\chi)\psi_{x,9} Y_{v,w}^{x,y,9} + v\pi_w Y_{1-v,w}^{x,y,8} + \gamma_{x-1,y} Y_{v,w}^{x-1,y,8} - Y_{v,w}^{x,y,8}\left(\gamma_{x,y} + \mu_{v,w} + \alpha_{x,y,8} + (1-v)\pi_w + \phi_8\right); x \in \{2,3\}$$

$$\frac{d}{dt}\left(Y_{v,w}^{4,y,8}\right) = (1-\chi)\xi_0\theta_y f_{4,y} A_{v,w}^{3} + (1-\chi)\xi_1 Y_{v,w}^{4,y,3} + \sum_{Z=0}^{Z=3}(1-\chi)\psi_{0,Z}\theta_y f_{4,y} A_{v,w}^{Z} + \sum_{Z=0}^{Z=3}(1-\chi)\psi_{x,Z} Y_{v,w}^{4,y,Z} + (1-\chi)\psi_{x,9} Y_{v,w}^{4,y,9} + v\pi_w Y_{1-v,w}^{4,y,8} + \gamma_3 Y_{v,w}^{3,y,8} - Y_{v,w}^{4,y,8}\left(\mu_{v,w} + \alpha_{4,y,8} + (1-v)\pi_w + \phi_8\right)$$

MSM dropped out of ART:

$$\frac{d}{dt}\left(Y_{v,w}^{1,y,9}\right) = \sum_{Z=4}^{Z=8}\phi_Z Y_{v,w}^{1,y,Z} + v\pi_w Y_{1-v,w}^{1,y,9} + \theta_y f_{x,y}\phi_4 A_{v,w}^{4} - Y_{v,w}^{1,y,9}\left(\gamma_{1,y} + \mu_{v,w} + \alpha_{1,y,9} + (1-v)\pi_w + \psi_{1,9} + \zeta\right)$$

$$\frac{d}{dt}\left(Y_{v,w}^{x,y,9}\right) = \sum_{Z=4}^{Z=8} \phi_Z Y_{v,w}^{x,y,Z} + v\pi_w Y_{1-v,w}^{x,y,9} + \theta_y f_{x,y} \phi_4 A_{v,w}^4 + \gamma_{x-1,y} Y_{v,w}^{x-1,y,9} - Y_{v,w}^{x,y,9}\left(\gamma_{x,y} + \mu_{v,w} + \alpha_{x,y,9} + (1-v)\pi_w + \psi_{x,9} + \zeta\right); x \in \{2,3\}$$

$$\frac{d}{dt}\left(Y_{v,w}^{4,y,9}\right) = \sum_{Z=4}^{Z=8} \phi_Z Y_{v,w}^{4,y,Z} + v\pi_w Y_{1-v,w}^{4,y,9} + \theta_y f_{x,y} \phi_4 A_{v,w}^4 + \gamma_3 Y_{v,w}^{3,y,9} - Y_{v,w}^{4,y,9}\left(\mu_{v,w} + \alpha_{4,y,9} + (1-v)\pi_w + \psi_{4,9} + \zeta\right)$$

Force of infection

$$\lambda_{v,w} = 1 - \left(\prod_{j=1}^{j=3} \prod_{v'=0}^{v'=1} \prod_{w'=0}^{w'=1} \left(\frac{\sum_{z=0}^{z=1}\left(X_{v\prime,w\prime}^{z}\right)}{N_{v\prime,w\prime}} + \frac{\sum_{z=0}^{z=3}\left(A_{v\prime,w\prime}^{z}\right)}{N_{v\prime,w\prime}} \left(1 - d_1\beta\left(1 - e_c s_{c,j}\right)(1 - e_n s_n)\right)^{n_j} + \sum_{y=1}^{y=4} \left(\frac{\sum_{x=1}^{x=3}\left(\sum_{z=0}^{z=3}\left(Y_{v\prime,w\prime}^{x,y,z}\right) + Y_{v\prime,w\prime}^{x,y,8} + Y_{v\prime,w\prime}^{x,y,9}\right)}{N_{v\prime,w\prime}} \left(1 - h_y\beta\left(1 - e_c s_{c,j}\right)(1 - e_n s_n)\right)^{n_j} \right) + \sum_{y=1}^{y=4} \left(\frac{\left(\sum_{z=0}^{z=3}\left(Y_{v\prime,w\prime}^{4,y,z}\right) + Y_{v\prime,w\prime}^{4,y,8} + Y_{v\prime,w\prime}^{4,y,9}\right)}{N_{v\prime,w\prime}} \left(1 - d_2 h_y\beta\left(1 - e_c s_{c,j}\right)(1 - e_n s_n)\right)^{n_j} \right) + \frac{A_{v\prime,w\prime}^{4}}{N_{v\prime,w\prime}} \left(1 - d_3\beta\left(1 - e_c s_{c,j}\right)(1 - e_n s_n)\right)^{n_j} + \sum_{y=1}^{y=4} \left(\frac{\sum_{x=1}^{x=3}\left(Y_{v\prime,w\prime}^{x,y,4}\right)}{N_{v\prime,w\prime}} \left(1 - d_4 h_y\beta\left(1 - e_c s_{c,j}\right)(1 - e_n s_n)\right)^{n_j} \right) + \sum_{y=1}^{y=4} \left(\frac{\left(Y_{v\prime,w\prime}^{4,y,4}\right)}{N_{v\prime,w\prime}} \left(1 - d_5 h_y\beta\left(1 - e_c s_{c,j}\right)(1 - e_n s_n)\right)^{n_j} \right) + \sum_{y=1}^{y=4} \left(\frac{\sum_{x=1}^{x=4}\sum_{z=5}^{z=7}\left(Y_{v\prime,w\prime}^{x,y,z}\right)}{N_{v\prime,w\prime}} \left(1 - d_6 h_y\beta\left(1 - e_c s_{c,j}\right)(1 - e_n s_n)\right)^{n_j} \right) \right)^{\rho_{vw,v\prime w\prime,j} c_{v,w,j}} \right)$$

where the total number of MSM partners in age group v' and race group w' is calculated as:

$$N_{v',w'} = \sum_{z=0}^{z=1} \left(X_{v',w'}^{z}\right) + \sum_{z=0}^{z=4} \left(A_{v',w'}^{z}\right) + \sum_{x=1}^{x=4} \sum_{y=1}^{y=4} \sum_{z=0}^{z=9} \left(Y_{v',w'}^{x,y,z}\right)$$

Infection risk is estimated for three partner types (j = 1: regular partners, j = 2: casual partners; j = 3: commercial partners). e_c is per-sex-act condom efficacy, $s_{c,j}$ is the proportion of sex acts in which a condom is used with partners of type j, e_n is per-sex act reduction in HIV acquisition risk due to male circumcision, and s_n is the proportion of MSM who are circumcised. β is the average probability of acquiring HIV infection from an anal sex act with an HIV-positive male partner with chronic infection and CD4>200 cells/µl who is not taking ART, $\rho_{vw,v'w',j}$ is, for MSM in age group v and race group w, the proportion of partners of type j who are in age group v' and race group w' . $c_{v,w,j}$ is the average number of new partners per year of type j for MSM in age group v and race group w, n_j is the average number of sex acts per partnership for a partnership of type j, $d_1$is the relative infectiousness of those in the acute versus chronic stage of infection, d_2 is the relative infectiousness of those with CD4<200 cells/µl versus those with chronic infection and CD4>200 cells/µl, d_3, d_4, d_5 are the relative infectiousness of those on ART with a partially suppressed viral load who have acute infection, chronic infection (CD4>200 cells/µl) or CD4<200 cells/µl, respectively, versus those untreated with chronic infection and CD4>200 cells/µl , $d_6$is the relative infectiousness of those on ART with a fully suppressed viral load versus those untreated with chronic infection and CD4>200 cells/µl, and h_y is the relative infectiousness of those not fully virally suppressed who have SPVL y.

The relative infectiousness of those on ART with a partially suppressed viral load are calculated as follows:

$$d_3 = d_6 + d_r(d_1 - d_6)$$

$$d_4 = d_6 + d_r(1 - d_6)$$

$$d_5 = d_6 + d_r(d_2 - d_6)$$

Where d_r is the relative level of infectiousness of those partially suppressed, scaled between the level for those fully suppressed ($d_r = 0$).and those unsuppressed ($d_r = 1$).

Numerical integration of differential equations

For the Runge Kutta method used to solve the equations (StepperDopr853 code from Press et al 2007 - Numerical Recipes: the Art of Scientific Computing, 3rd Edition), the initial stepsize (h1) was set to 0.01 years, the minimum stepsize (hmin) to 0.0 years, with both the absolute and relative error (atol, rtol) set to 1 x 10^{-6}.

Model parameters

Demographic parameters describing the initial age- and race-composition of the MSM population in 1984, and the age-and race-composition of new MSM entering the population, were estimated from NHBS and census data. Non-HIV related age- and race-specific death rates came from CDC data for Maryland state[2]. Following trends in Baltimore City census data, we assume the MSM population has declined at a similar rate as the total population from 1980 until 2010, primarily due to out-migration of white residents[3]. Rates of in- and out-migration were estimated to fit demography data.

Sexual behaviour parameters including age- and race-specific numbers of new main, casual and commercial anal sex partners per year, condom use in each type of partnership, circumcision status, and sexual mixing by age and race, were estimated from NHBS surveys.

The number of sex acts in main and casual partnerships were estimated from published studies of US MSM[4-6].

Based upon trends in NHBS data, condom use is assumed to increase over time up until 2008, and then to decrease (or, in a small proportion of runs, to increase) between 2008 and 2011, staying constant after 2011. Condom use in main partnerships is higher for black than white MSM (59% vs 36%), and higher in casual partnerships (67%). At the beginning of the epidemic, condom use is assumed to be fixed at a certain level for all partnership types.

The number of new casual and commercial partners is assumed to decline linearly over time from 1984 until 2011, subsequently staying constant, consistent with NHBS and historical data[7], while the number of new main partners is assumed to have stayed the same over time in line with NHBS data. In 2011, the greatest numbers of new partners were reported by black MSM aged 18-24, and the fewest by white MSM aged 18-24 (table 1). Note that behavioural parameters (partner numbers and condom use) are assumed to be the same among those who never test for HIV and those who do.

Mixing parameters (by age and race) were estimated from 2008 NHBS data on the percentage of partners of black and white MSM who were black or white, and 2011 NHBS data on the

age and race of partners by age and race (N.B. the age-group of partners could only be estimated for MSM aged 18-24). Assuming that the proportion of people in each age and race group in the survey was representative of the wider MSM population, and using the overall number of anal sex partners per year reported by each group, least-squares fitting was used to identify the most likely values for the mixing parameters by age and race. The data suggested some preference for partners of a similar age and strong preference for partners of the same race.

Disease progression parameters by SPVL, transmission probabilities and intervention efficacies (for condoms, circumcision, and HIV treatment) were obtained from published studies[8-18].

PrEP use was not modelled as very low levels of use were reported in the 2014 NHBS survey.

The proportion never testing was estimated from the proportion of MSM in NHBS 2004-2011 surveys aged >24 years old who report never having had an HIV test. This was not stratified by race.

For care continuum parameters, age- and race specific HIV testing rates were estimated from NHBS data for 2004-2011 - as no clear trend was seen over these surveys, testing rates were assumed to remain constant after 2004 (ranging from 51% (>24 year old white men) to 79% (18-24 year old black men) testing each year. The overall percentage of MSM testing in the last year was estimated at 25% for MSM in a national survey in 1996 [19], and so the percentage testing in the last 12 months is assumed to have increased linearly over time prior to 2004 to give the overall percentage consistent with this data in 1996. The percentage testing in the last year was converted into an annual testing rate for use in the model.

Similarly, race-specific percentages linking to care immediately after diagnosis was estimated from DH data for Baltimore, national surveys and US national CDC data on the proportion linking to care within three months of diagnosis, and assumed to increase linearly until 2008 (in line with US national CDC data), then stay constant (at 81% for white MSM, 70% for black MSM [20-26]).

Historical US ART guidelines were used to determine at which CD4 count ART initiation occurred between 1998 and 2012, when universal ART was recommended.

Rates of ART initiation due to development of AIDS symptoms were based upon CD4-count specific rates of incidence of AIDS-defining illness (following CDC definitions of AIDS-defining illness)[39,40]; we assumed that those developing AIDS symptoms would rapidly seek care and be put onto ART.

Data from MSM in the Johns Hopkins HIV cohort (Baltimore) who initiated ART after 1/1/2005 with VL>1000 copies/ml (N=251), and data from NA-ACCORD from Fenway electronic monitoring data (Boston) for patients who achieved viral suppression and had VL>200 copies/ml at ART initiation (N=998) were used to estimate median time to viral suppression (defined as VL<200 copies/ml) stratified by initial viral load (log10VL<4.0,4.0-4.5,4.5-5.0.>5.0).

Other care continuum parameters including race-related rates of ART initiation, race-related rates of dropout from care and ART and ART adherence by race were taken from published studies among US populations, where possible studies of MSM[20, 24, 27-31]. ART adherence was based upon estimates of the proportion of PLHIV achieving viral suppression. Estimates and sources for all parameters are given in Table S1.

Model calibration

The model was fitted to MSM population size, age and race distribution, HIV prevalence by age and race, percentage of HIV-positive MSM diagnosed, percentage of diagnosed MSM in care and percentage virally suppressed, ART coverage and percentage on ART virally suppressed (table 1, table S2). Model predictions were accepted as fits if they fell within designated ranges for these quantities at all time-points before 2014 (HIV prevalence had to fall within range at 2 out of 3 time-points).

Addressing uncertainties across data sources

First, the race and particularly the age distribution of MSM in the NHBS samples differed from that of the (older) general male population in census data. The NHBS data are collected from MSM recruited from MSM-identified venues, meaning that they may not reflect the demography of the wider MSM population (e.g. older MSM may be less likely to attend MSM-identified venues). While in the absence of migration we would expect the demography of the MSM population to be similar to the demography of the general male population (as captured by the census) it is possible that migration could affect this – for example, black MSM in the Baltimore NHBS report having lived in the city for longer than white MSM, suggesting differential migration patterns by race, which could affect the demography of the local MSM population. Given uncertainty about the representativeness of the NHBS sample, we separately fitted the model under two demography assumptions fitting to either (a) the ***NHBS age/race distribution*** or (b) the ***census age/race distribution*** of the general male population (using lower rates of population entry and exit). Second, estimates of the annual HIV testing rate parameter obtained from NHBS data produced higher predictions of the percentage of MSM diagnosed than CDC estimates for MSM in Maryland, so we separately fitted the model under two diagnosis assumptions either (c) using ***NHBS HIV testing rate*** parameters (without fitting to CDC diagnosis data since it was not possible to do both) or (d) fitting the percentage of HIV-positive MSM diagnosed predicted by the model to ***CDC estimates for Maryland*** (allowing HIV testing rate parameters to take lower values than suggested by the NHBS data). Similarly and finally, we found that fitting to ART coverage data estimated from NHBS plasma testing suggested higher levels of viral suppression than fitting to Maryland DH data on the percentage of diagnosed MSM who are virally suppressed (and the percentage in care), and so the model was also fitted separately under two care continuum assumptions, either fitting to (e) ***NHBS ART coverage data*** or (f) ***DH continuum data.*** Note that the plasma measures of ART used in the NHBS sub-study are indicative of ART use in the past 24 hours, not longer-term use, so we have conservatively assumed that this is indicative of the total number of MSM on ART, not only those who are fully adherent, and have fitted the proportion of all MSM on ART to this NHBS ART coverage estimate. The 3 groups of fitting assumptions (demography, diagnosis, care continuum), with 2 assumptions each, resulted in 8 combinations of assumptions to fit to (table S2).

Table S1. Parameters used in the HIV transmission model, with source and justification

Symbol	Parameter	Range of values	Source/justification
INITIAL CONDITIONS			
N_0	Initial size of MSM population (1984)	6765-8326	260,199 men aged 18+ in the 1980 Baltimore census; Purcell et al. 2012[32] estimate % of US men had same-sex behaviour last 12 months 2.9% (95% CI 2.6-3.2%)
	Percentage of MSM who were black in 1984	50-64	Main estimate: overall population 1980 census. Upper limit: MSM in NHBS 2004; lower limit: lower 95% CI in NHBS 2004
	Percentage of black MSM aged 18-24 in 1984	16-31	Lower bound: black men in 2010 census<br>Upper bound: black MSM NHBS 2004 (upper 95% CI)
	Percentage of white MSM aged 18-24 in 1984	14-28	Lower bound: white men in 2010 census<br>Upper bound: white MSM NHBS 004 (upper 95% CI)
	HIV prevalence among black MSM in 1984 (%)	15-44	MACS baseline black MSM [33]– lower bound a third of this as non-random sample
	HIV prevalence among white MSM in 1984 (%)	9-28	MACS baseline white MSM [33]– lower bound a third of this as non-random sample
Demography			
Γ	Rate at which new MSM join the sexually active MSM population (per year)	100-400 (fitting to census demography)<br>200-800 (fitting to NHBS demography)	estimate
m_{black}	Percentage of new incoming MSM who are black	60-85	Baltimore census 1990-2010; NHBS 2004-2011
$m_{young,0}$	Percentage of new incoming black MSM who are aged 18-24 years old	72-87	% of black MSM in NHBS who say they entered sexually active Baltimore MSM population aged <25 – 2008 & 2011 NHBS
$m_{young,1}$	Percentage of new incoming white MSM who are aged 18-24 years old	50-71 (fitting to census demography)<br>37-71 (fitting to NHBS demography)	% of white MSM in NHBS who say they entered sexually active Baltimore MSM population aged <25 – 2008 & 2011 NHBS
π_0	rate of moving from 18-24 year old age group to >24 year old age group, black MSM, per year	0.17 (fixed)	Mean age at joining the local MSM population in NHBS 2008 and 2011 for 18-24 yr old MSM ~16 yrs old (95% CI 15-17)
π_1	rate of moving from 18-24 year old age group to >24 year old age group, white MSM, per year	0.17-0.25	Mean age at joining the local MSM population in NHBS 2008 and 2011 for 18-24 yr old MSM ~18/19 yrs old (95% CI 16/17-20)

$\mu_{0,0}$	Non-HIV related death rate, 18-24 year old black men, per year	0.0011-0.0015	CDC WONDER database data for Maryland; data for 15-24 years olds
$\mu_{1,0}$	Non-HIV related death/leaving rate, >24 year old black men, per year	0.011-0.04 (census fitting)<br>0.041-0.11 (NHBS fitting)	CDC WONDER database data for Maryland; average death rate over ages 26-64 years old<br>Upper bound: add on 1/36 (double current duration as an MSM)<br>NHBS fitting: additionally assume extra rate of ceasing to attend NHBS venues
$\mu_{0,1}$	Non-HIV related death rate, 18-24 year old white men, per year	0.00075-0.001	CDC WONDER database data for Maryland; data for 15-24 years olds
$\mu_{1,1}$	Non-HIV related death/leaving rate, >24 year old white men, per year	0.033-0.1 (census fitting)<br>0.058-0.128 (NHBS fitting)	High rates reflecting out-migration plus rates of ceasing sexual activity;<br>NHBS fitting: additionally assume extra rate of ceasing to attend NHBS venues
Sexual behaviour			
n_1	Number of sex acts per main partnership	40-470	48.2-85.1 sex episodes/year with main partners [4], partnerships last 3.5-5.5 years [5, 6], but assume can be shorter (~1 year)
n_2	Number of sex acts per casual partnership	1.5-6	3-4.9 sex episodes/year [4], partnerships last 0.5-1.3 years [5]
n_3	Number of sex acts per commercial partnership	1-2	assumed
$c_{0,0,1}$	Number of new main partners per year, 18-24 year old black MSM	0.58-0.8	NHBS 2004, 2008, 2011
$c_{0,0,2}$	Number of new casual partners per year, 18-24 year old black MSM 2011 onwards‡	1.54-2.09	NHBS 2011
$c_{0,0,3}$	Number of new commercial partners per year, 18-24 year old black MSM 2011 onwards ‡	0-1.36	NHBS 2011
$c_{1,0,1}$	Number of new main partners per year, >24 year old black MSM	0.36-0.57	NHBS 2004, 2008, 2011
$c_{1,0,2}$	Number of new casual partners per year, >24 year old black MSM 2011 onwards ‡	0.81-1.24	NHBS 2011
$c_{1,0,3}$	Number of new commercial partners per year, >24 year old black MSM 2011 onwards ‡	0.15-0.85	NHBS 2011
$c_{0,1,1}$	Number of new main partners per year, 18-24 year old white MSM	0.08-0.37	NHBS 2004, 2008, 2011
$c_{0,1,2}$	Number of new casual partners per year, 18-24 year old white MSM 2011 onwards ‡	0.05-0.93	NHBS 2011

$c_{0,1,3}$	Number of new commercial partners per year, 18-24 year old white MSM 2011 onwards ‡	0-0.28	NHBS 2011
$c_{1,1,1}$	Number of new main partners per year, >24 year old white MSM	0.11-0.21	NHBS 2004, 2008, 2011
$c_{1,1,2}$	Number of new casual partners per year, >24 year old white MSM 2011 onwards ‡	0.28-1.07	NHBS 2011
$c_{1,1,3}$	Number of new commercial partners per year, >24 year old white MSM 2011 onwards ‡	0-0.07	NHBS 2011
Partner_number_decline	absolute decline per year in the number of new casual or commercial partners	0.17-0.36	From trends in NHBS data on number of commercial and causal partners 2004-2011
Mixing_param__age	Scale between fully proportionate and fully assortative mixing by age	0.25-0.35	estimated from NHBS 2011 data on last partner
Mixing_param_race	Scale between fully proportionate and fully assortative mixing by race	0.7-0.8	0.75 estimated from NHBS 2011 data on last partner and 0.74 from NHBS additional data 2008 [34]
Early_condom_use	Minimum level of condom use at start of the HIV epidemic (% of sex acts)	0-30	No data
$s_{c,1,0}$	Percentage of sex acts in which a condom is used, main partnerships where both partners are black, 2004 onwards ‡	47-67	condom use last sex act reported by black MSM with main partners NHBS 2004-2011
$s_{c,1,1}$	Percentage of sex acts in which a condom is used, main partnerships where one or both partners are white, 2004 onwards ‡	30-39	condom use last sex act reported by white MSM with main partners NHBS 2004-2011
$s_{c,2}$	Percentage of sex acts in which a condom is used, casual partnerships (any race partner), 2004 onwards ‡	63-72	condom use last sex act reported in casual partnerships NHBS 2004-2011
$s_{c,3}$	Percentage of sex acts in which a condom is used, commercial partnerships (any race partner), 2004 onwards ‡	21-78	condom use last sex act reported in commercial partnerships NHBS 2004 & 2008
Condom_change_1	Yearly change in % of sex acts in which condoms are used, all partnerships prior to 2008	2.4-4	From trend in data from NHBS 2004-2008, averaging over condom use in main and casual partnerships

Condom _change _2	Yearly change in % of sex acts in which condoms are used, all partnerships between 2008 and 2011	-2.4-+0.2	From trends in data from NHBS 2008-2011 and 2008-2014, averaging over condom use in main and casual partnerships
HIV disease progression			
$1/\gamma_a$	Average duration of acute infection, months	2-6	[11, 35]
$\alpha_{0,0,z}$	HIV-related death rate for those with acute HIV infection, per year	0 (fixed)	assumption
$\alpha_{1,y,0}$ $\alpha_{1,y,1}$ $\alpha_{1,y,2}$ $\alpha_{1,y,3}$ $\alpha_{1,y,8}$ $\alpha_{1,y,9}$	HIV-related death rate for those with CD4>500, off ART, per year	0.0009-0.0054	aged 25-44 in the European CASCADE cohort [9]; general population death rate subtracted
$\alpha_{2,y,0}$ $\alpha_{2,y,1}$ $\alpha_{2,y,2}$ $\alpha_{2,y,3}$	HIV-related death rate for those with CD4 350-500, off ART, per year	0.0009-0.0069	aged 25-44 in the European CASCADE cohort [9]; general population death rate subtracted
$\alpha_{3,y,1}$ $\alpha_{3,y,1}$	HIV related death rate for those with CD4 200-350, off ART, per year	0.0045-0.0135	aged 25-44 in the European CASCADE cohort [9]; general population death rate subtracted
$1/\alpha_{4,1,1}$	Inverse of HIV-related death rate for those with CD4<200, SPVL<4.0, off ART (years)	3.28-12.87	Netherlands ATHENA cohort [8]
$1/\alpha_{4,2,1}$	Inverse of HIV-related death rate for those with CD4<200, SPVL 4.0-4.5, off ART (years)	1.43-6.09	Netherlands ATHENA cohort [8]
$1/\alpha_{4,3,1}$	Inverse of HIV-related death rate for those with CD4<200, SPVL 4,5-5,0, off ART (years)	4.41-23.64	Netherlands ATHENA cohort [8]
$1/\alpha_{4,4,1}$	Inverse of HIV-related death rate for those with CD4<200, SPVL>5.0, off ART (years)	1.32-3.59	Netherlands ATHENA cohort [8]
$\alpha_{1,y,4}$, $\alpha_{2,y,4}$ $\alpha_{1,y,5}$, $\alpha_{2,y,5}$	HIV-related mortality for those with CD4>500 or CD4 350-500 at start of treatment, for 1st , 2nd and subsequent years on ART, per year	0-0.003	From probabilities for those with CD4>350 [36]; general population death rate subtracted [37]

$\alpha_{1,y,6}$, $\alpha_{2,y,6}$ $\alpha_{1,y,7}$ $\alpha_{2,y,7}$			
a_1	Relative mortality of those with CD4 200-350 vs CD4>350 at start of treatment, 1st year on ART	1.2-2.8	[10]
a_2	Relative mortality of those with CD4 200-350 vs CD4>350 at start of treatment, 2nd year on ART	1-2.2	[10] Upper limit reduced to give main estimate as midpoint
a_3	Relative mortality of those with CD4 200-350 vs CD4>350 at start of treatment, 3rd year + on ART	1-1.4	[10] Upper limit reduced to give main estimate as midpoint
a_4	Relative mortality of those with CD4 <200 vs CD4>350 at start of treatment, 1st year on ART	1.8-5.2	[10] Main estimate and lower bound: CD4 100-199; upper bound from those with CD4 25-49
a_5	Relative mortality of those with CD4 <200 vs CD4>350 at start of treatment, 2nd year on ART	1.3-6.2	[10] Main estimate and lower bound: CD4 100-199; upper bound from those with CD4 25-49
a_6	Relative mortality of those with CD4 <200 vs CD4>350 at start of treatment, 3rd year + on ART	1-3.2	[10] Main estimate and lower bound: CD4 100-199; upper bound from those with CD4 50-99
b_1	Relative mortality of those with AIDS before ART initiation vs without, 1st year on ART	3.0-4.8	[10]
b_2, b_3	Relative mortality of those with AIDS before ART initiation vs without, 2nd , 3rd + years on ART	1.4-2.6	[10]
k_4	Percentage of those starting ART with CD4<200 who have a prior AIDS diagnosis	40-60	[38]
θ_2	Percentage of HIV-positive MSM with a SPVL 4.0-4.5	25 (fixed)	Netherlands ATHENA cohort [8]; US MSM (MACS cohort)[41, 42]
θ_3	Percentage of HIV-positive MSM with a SPVL 4.5-5.0	25-40	Netherlands ATHENA cohort [8]; US MSM (MACS cohort)[41, 42]
θ_4	Percentage of HIV-positive MSM with a SPVL >5.0	10-25	Netherlands ATHENA cohort [8]; US MSM (MACS cohort)[42]

$1/\gamma_{1,1}$	Average duration spent with CD4>500 cells/µl, for those with SPVL <4.0 (years)	4.56-6.37	Netherlands ATHENA cohort [8]
$1/\gamma_{2,1}$	Average duration spent with CD4 350-500, for those with SPVL <4.0 (years)	2.98-4.53	Netherlands ATHENA cohort [8]
$1/\gamma_{3,1}$	Average duration spent with CD4 200-350, for those with SPVL <4.0 (years)	5.04-13.69	Netherlands ATHENA cohort [8]
$1/\gamma_{1,2}$	Average duration spent with CD4>500, for those with SPVL 4.0-4.5 (years)	2.68-3.64	Netherlands ATHENA cohort [8]
$1/\gamma_{2,2}$	Average duration spent with CD4 350-500, for those with SPVL 4.0-4.5 (years)	2.65-3.64	Netherlands ATHENA cohort [8]
$1/\gamma_{3,2}$	Average duration spent with CD4 200-350, for those with SPVL 4.0-4.5 (years)	5.46-15.55	Netherlands ATHENA cohort [8]
$1/\gamma_{1,3}$	Average duration spent with CD4>500, for those with SPVL 4.5-5.0 (years)	2.08-2.64	Netherlands ATHENA cohort [8]
$1/\gamma_{2,3}$	Average duration spent with CD4 350-500, for those with SPVL 4.5-5.0 (years)	1.98-2.72	Netherlands ATHENA cohort [8]
$1/\gamma_{3,3}$	Average duration spent with CD4 200-350, for those with SPVL 4.5-5.0 (years)	4.73-10.22	Netherlands ATHENA cohort [8]
$1/\gamma_{1,4}$	Average duration spent with CD4>500, for those with SPVL ≥5.0 (years)	1.28-1.76	Netherlands ATHENA cohort [8]
$1/\gamma_{2,4}$	Average duration spent with CD4 350-500, for those with SPVL ≥5.0 (years)	1.22-1.69	Netherlands ATHENA cohort [8]
$1/\gamma_{3,4}$	Average duration spent with CD4 200-350, for those with SPVL ≥5.0 (years)	2.12-4.19	Netherlands ATHENA cohort [8]
$1/\sigma_0$	Average duration from ART initiation to viral suppression (VL < 200 copies/ml) for those with acute HIV infection (months)	3.93-8.50	Pregnant women, Kenya[43]
$1/\sigma_1$	Average duration from ART initiation to viral suppression (VL < 200 copies/ml) for those with $\log_{10}$ SPVL <4.0 (months)	0.95-4.1	Data from Johns Hopkins (Baltimore) and Fenway (Boston); estimate is weighted average of median values from 2 sites
$1/\sigma_2$	Average duration from ART initiation to viral suppression (VL < 200 copies/ml) for those with $\log_{10}$ SPVL 4.0-4.5 (months)	1.03-4.75	Data from Johns Hopkins (Baltimore) and Fenway (Boston); estimate is weighted average of median values from 2 sites

$1/\sigma_3$	Average duration from ART initiation to viral suppression (VL < 200 copies/ml) for those with $\log_{10}$ SPVL 4.5-5.0 (months)	1.4-6.43	Data from Johns Hopkins (Baltimore) and Fenway (Boston); estimate is weighted average of median values from 2 sites
$1/\sigma_4$	Average duration from ART initiation to viral suppression (VL < 200 copies/ml) for those with $\log_{10}$ SPVL >5.0 (months)	2.03-6.49	Data from Johns Hopkins (Baltimore) and Fenway (Boston); estimate is weighted average of median values from 2 sites
$f_{1,1}$	Percentage with CD4 >500 after seroconversion, for those with SPVL <4.0	81-91	Netherlands ATHENA cohort [8]
$f_{3,1}$	Percentage with CD4 200-350 after seroconversion, for those with SPVL <4.0	0-4	Netherlands ATHENA cohort [8]
$f_{4,1}$	Percentage with CD4 <200 after seroconversion, for those with SPVL <4.0	0 (fixed)	Netherlands ATHENA cohort [8]
$f_{1,2}$	Percentage with CD4 >500 after seroconversion, for those with SPVL 4.0-4.5	72-83	Netherlands ATHENA cohort [8]
$f_{3,2}$	Percentage with CD4 200-350 after seroconversion, for those with SPVL 4.0-4.5	1-5	Netherlands ATHENA cohort [8]
$f_{4,2}$	Percentage with CD4 <200 after seroconversion, for those with SPVL 4.0-4.5	0 (fixed)	Netherlands ATHENA cohort [8]
$f_{1,3}$	Percentage with CD4 >500 after seroconversion, for those with SPVL 4.5-5.0	69-79	Netherlands ATHENA cohort [8]
$f_{3,3}$	Percentage with CD4 200-350 after seroconversion, for those with SPVL 4.5-5.0	3-8	Netherlands ATHENA cohort [8]
$f_{4,3}$	Percentage with CD4 <200 after seroconversion, for those with SPVL 4.5-5.0	0 (fixed)	Netherlands ATHENA cohort [8]
$f_{1,4}$	Percentage with CD4 >500 after seroconversion, for those with SPVL ≥5.0	64-77	Netherlands ATHENA cohort [8]
$f_{3,4}$	Percentage with CD4 200-350 after seroconversion, for those with SPVL ≥5.0	2-7	Netherlands ATHENA cohort [8]
$f_{4,4}$	Percentage with CD4 <200 after seroconversion, for those with SPVL ≥5.0	0 (fixed)	Netherlands ATHENA cohort [8]
$r_{y,Y}$	Percentage going into each SPVL compartment after dropping out of ART		Stay in same SPVL compartment after dropping out of ART
$q_{x,X,Z}$	Percentage going from each starting CD4 count to new CD4 compartment upon dropping out of ART		Assume CD4 count when drop out of ART same as when started ART

Transmission probabilities			
d_1	Relative infectiousness of HIV-positive partner in acute stage of infection vs chronic & CD4>200 (off ART)	4.47-18.81	[35]
d_2	Relative infectiousness of HIV-positive partner in late stage of infection – CD4<200 cells/µl vs chronic and CD4>200 (off ART)	2-8	[44, 45]
β	Average probability of acquiring HIV infection per sex act with an HIV-positive partner with chronic untreated infection	0.0007-0.0285	[13, 14]; assume 50% of sex acts are insertive
h_1	Relative infectiousness of HIV-positive person with $\log_{10}$ SPVL <4.0 vs 4.0-4.5	0.337-0.68	[16] Inverse of pooled increase in transmissibility per log10 decrease in viral load
h_2	Relative infectiousness of HIV-positive person with $\log_{10}$ SPVL 4.0-4.5 vs 4.0-4.5	1 (fixed)	
h_3	Relative infectiousness of HIV-positive person with $\log_{10}$ SPVL 4.5-5.0 vs 4.0-4.5	1 (fixed)	
h_4	Relative infectiousness of HIV-positive person with $\log_{10}$ SPVL >5.0 vs 4.0-4.5	1.47-2.97	[16] pooled increase in transmissibility per log10 increase in viral load
Intervention behaviour			
p	Percentage of new entrants to MSM population who never routinely test for HIV	5-13	NHBS Baltimore MSM 2004-2011: % of those aged >24 years old who report never testing for HIV
$\tau_{0,0}$	Percentage of undiagnosed black MSM aged 18-24 testing for HIV in the last year, 2004 onwards ‡	63.8-95.0 (reported testing rates)<br>25.5-47.5 (diagnosis fitting)	NHBS data 2004-2011, self-reported HIV negative men; converted into rate of testing at least once per year in the model<br>Diagnosis fitting: 60% reduction
$\tau_{0,1}$	Percentage of undiagnosed white MSM aged 18-24 testing for HIV in the last year, 2004 onwards ‡	32.1-82.3 (reported testing rates)<br>12.8-41.2 (diagnosis fitting)	NHBS data 2004-2008(highest and lowest from ranges), self-reported HIV negative men<br>Diagnosis fitting: 60% reduction
$\tau_{1,0}$	Percentage of undiagnosed black MSM aged >24 years old testing for HIV in the last year, 2004 onwards ‡	50.0-70.2 (reported testing rates)<br>20.0-35.1 (diagnosis fitting)	NHBS data 2004-2011 (highest and lowest from ranges), self-reported HIV negative men<br>Diagnosis fitting: 60% reduction

$\tau_{1,1}$	Percentage of undiagnosed white MSM aged >24 years old testing for HIV in the last year, 2004 onwards ‡	32.7-69.7 (reported testing rates)<br>13.1-34.9(diagnosis fitting)	NHBS data 2004-2011 (highest and lowest from ranges), self-reported HIV negative men<br>Diagnosis fitting: 60% reduction
τ_{early}	Percentage of all MSM who tested for HIV in the last year, 1996	20-30 (reported testing rates)<br>8-15 (diagnosis fitting)	MSM in national NHSDA survey 1996 [19]<br>Diagnosis fitting: 60% reduction
ω	Ratio of rate of dropout from care: rate of dropout from ART	1-7	Estimates from US studies - risk of dropout from care for those on vs off ART [27, 46, 47]
q_1	Percentage of white MSM testing positive for HIV who link to care straight away	72-86	[20-24] From estimates of linkage to care within three months of HIV diagnosis.
ϵ	Rate of linkage to care for those not linking immediately or dropped out, per year	0-0.1 (fitting to care and viral suppression data)<br>0-0.2 0-0.5 (fitting to ART coverage data)	Estimate
linkage_inc	Annual absolute increase in percentage of white MSM who link to care straight away after testing positive for HIV	3.5 (fixed)	From changes for MSM in national CDC data [25, 26]
χ	Percentage of white MSM initiating ART who are adherent (achieve viral suppression)	73-99	[24, 29, 30, 48] From estimates of the proportion of PLHIV achieving viral suppression in mutli-site US cohorts and surveillance
ξ_x	Rate of initiation onto ART from care, when meeting CD4 criteria†, per year ‡	0.5-2.1 (fitting to care and viral suppression data)<br>1.1-4 (fitting to ART coverage data)	Assuming CD4 testing every 3-6 months (national guidelines), acceptance 80-90% [49]
$\psi_{0,z}, \psi_{1,z}$	Rate of starting HAART due to AIDS symptoms, CD4>500, per year (post-1996)	0.002-0.01	Incidence of AIDS-defining illness among ART naives, CASCADE collaboration [39]; similar estimates from EURO-COORD data analysis [40]
$\psi_{2,z}$	Rate of starting HAART due to AIDS symptoms, CD4 350-500, per year (post-1996)	0.008-0.015	Incidence of AIDS-defining illness among ART naives, CASCADE collaboration [39]; similar estimates from EURO-COORD data analysis [40]

$\psi_{3,z}$	Rate of starting HAART due to AIDS symptoms, CD4 200-350, per year (post-1996)	0.018-0.032	Incidence of AIDS-defining illness among ART naives, CASCADE collaboration [39]; similar estimates from EURO-COORD data analysis [40]
$\psi_{4,z}$	Rate of starting HAART due to AIDS symptoms, CD4<200, per year (post-1996)	0.173-0.262	Incidence of AIDS-defining illness among ART naives, CASCADE collaboration [39]
$\phi_4\phi_5\phi_6$	Dropout from ART, not fully suppressed/1st year on ART/2nd year on ART, per year	0.06-0.13	Rate of dropout from ART, US [28] [46] [27, 47],[50]
$\phi_{z\ ratio}$	Ratio of dropout from ART 3rd+ years: dropout 1st, 2nd years (ϕ_7: ϕ_4)	0.5-1.0	Rate of dropout from US ART cohorts [31]
ζ	Rate of re-enrolment into pre-ART HIV care for those dropping out of ART, per year	0.05-1	From rate of dropout and re-joining US ART cohorts [31]
s_n	Percentage of MSM circumcised	77-89	NHBS 2008 & 2011
ϵ_{ratio}	Ratio of rates of linkage to care for black:white MSM (ratio also applied to percentage linking immediately after diagnosis)	1-2 (fitting to care and viral suppression data) 0.84-1.5 (fitting to ART coverage data)	[20, 24] From estimates of linkage to care within three months of HIV diagnosis.
ω_{ratio}	Ratio of dropout from care for white:black MSM	1-3 (fitting to care and viral suppression data) 0.46-1.54 (fitting to ART coverage data)	[27, 47, 51]
ξ_{ratio}	Ratio of ART initiation rate for black:white MSM	0.4-1.0	[27]
$\phi_{w\ ratio}$	Ratio of ART dropout for black:white MSM	0.7-1.6	[28, 31]
χ_{ratio}	Ratio of percentage adherent to ART black:white MSM	0.82-1	[24, 29, 30, 46, 48, 52] From estimates of the proportion of PLHIV achieving viral suppression in multi-site US cohorts and surveillance
Intervention efficacy			
e_c	Per-sex-act reduction in HIV acquisition risk due to correct condom use (%)	58-79	Estimate for US MSM [17]
e_n	Per-sex-act reduction in HIV acquisition risk due to male circumcision (%)	12-23	Assuming same efficacy as for heterosexual men from RCTs [18], only protective in insertive acts, half of all sex acts are insertive, receptive sex acts carry a 2.3x higher risk of transmission than insertive[14].
d_r	Relative level of infectiousness of those on ART and partially suppressed, scaled between the level	0.5(fixed)	assumption

	for those fully suppressed ($d_r = 0$).and those unsuppressed ($d_r = 1$)		
d_6	Per-sex-act reduction in HIV transmission risk when on ART and fully suppressed vs chronic infection untreated (CD4>200) (%)	99-100	Estimates from discordant MSM partnerships where HIV-positive partner on ART and virally suppressed [15]

†Guideline changes coded in: pre-1996, no initiation of ART [53]. From 1996-1998 ART initiation at any CD4 count; from 1998-Feb 2001, initiation from care with CD4<500 (1998 guidelines); from Feb 2001-Dec 2009 initiation with CD4 <350 (2001 guidelines); from Dec 2009-March 2012 initiation from care with CD4<500 (2009 guidelines); from March 2012 onwards initiation from care with any CD4 count (2012 guidelines). These apply to all age and race groups.

‡Final values for time-varying parameters. Earlier values or earlier gradient of parameter function given elsewhere in table S1.

Table S2: Data fitted to, with fitting bounds, source and justification

Output	Year	Estimate	Min	Max	Source & justification	Fitting assumption used for		Used for validation
**Demography**						NHBS age/race distribution	Census age/race distribution	
Total MSM population size	2010	6518	4270	8765	Range 1.9-3.9%[32] of male population aged 18+ in Baltimore 2010 census (224,742)	✓	✓	
Percentage of population aged 18-24	1990	15	10	20	Census estimate ± 5pp		✓	
	2000	15	10	20	Census estimate ± 5pp		✓	
	2010	16	11	21	Census estimate ± 5pp		✓	
	2004	24.8	20.3	30.0	NHBS data 95% CI	✓		
	2008	30.6	22.9	40.0	NHBS data 95% CI	✓		
	2011	30.9	21.5	42.2	NHBS data 95% CI	✓		
	2014	23.9	18.0	31.0	NHBS data 95% CI			✓
Percentage of white MSM aged 18-24	2010	14	9	19	Census estimate ± 5pp		✓	
	2004	21.0	15.6	27.6	NHBS data 95% CI	✓		
	2008	17.1	9.4	29.3	NHBS data 95% CI	✓		
	2011	20.7	11.8	33.7	NHBS data 95% CI	✓		
	2014	14.6	8.6	23.6	NHBS data 95% CI			✓
Percentage of black MSM aged 18-24	2010	16	11	21	Census estimate ± 5pp		✓	
	2004	24.0	18.0	31.2	NHBS data 95% CI	✓		
	2008	32.5	23.8	42.6	NHBS data 95% CI	✓		
	2011	34.3	22.3	48.7	NHBS data 95% CI	✓		
	2014	27.2	19.4	36.7	NHBS data 95% CI			✓
Percentage of MSM who are black	1990	61	56	66	Census estimate ± 5pp		✓	
	2000	68	63	73	Census estimate ± 5pp		✓	
	2010	69	64	74	Census estimate ± 5pp		✓	
	2004	64.1	49.8	76.3	NHBS data 95% CI	✓		
	2008	73.1	59.3	83.6	NHBS data 95% CI	✓		
	2011	84.2	71.6	91.8	NHBS data 95% CI	✓		
	2014	73.8	64.6	81.3	NHBS data 95% CI			✓
**HIV prevalence**						All fitting assumptions		
HIV prevalence black MSM aged 18-24 years old	2004	33.0	23.6	43.8	NHBS data 95% CI	✓		
	2008	31.2	20.6	44.2	NHBS data 95% CI	✓		
	2011	39.6	32.0	47.8	NHBS data 95% CI	✓		

	2014	24.1	14.5	37.1	NHBS data 95% CI			✓
HIV prevalence black MSM aged >24 years old	2004	58.4	46.7	69.3	NHBS data 95% CI	✓		
	2008	51.8	42.9	60.7	NHBS data 95% CI	✓		
	2011	52.2	44.1	60.3	NHBS data 95% CI	✓		
	2014	47.4	39.4	55.5	NHBS data 95% CI			✓
HIV prevalence white MSM aged 18-24 years old	2004		0	100	Numbers too small			
	2008	22.2	10.1	42.0	NHBS data 95% CI	✓		
	2011		0	100	Numbers too small			
	2014		0	100	Numbers too small			
HIV prevalence white MSM aged >24 years old	2004	16.7	10.7	25.0	NHBS data 95% CI	✓		
	2008	18.4	9.4	32.9	NHBS data 95% CI	✓		
	2011	19.6	12.2	29.8	NHBS data 95% CI	✓		
	2014	9.1	5.0	15.9	NHBS data 95% CI			✓
**Care continuum indicators**						NHBS HIV testing rate	CDC estimates for Maryland	
Percentage of HIV-positive MSM diagnosed	2012	75.9	71.7	80.5	CDC data for Maryland state[54] 95% CI		✓	
						NHBS ART coverage data	Maryland DH continuum data	
Percentage of all HIV-positive MSM on ART	2008	39.5	31.9	47.5	NHBS ARV detection analysis [55] 95% CI	✓		
	2011	55.4	48.0	62.6	NHBS ARV detection analysis 95% CI	✓		
	2014	70.3	61.6	77.7	NHBS ARV detection analysis 95% CI			✓
Percentage of black HIV-positive MSM on ART	2008	36.9	28.5	46.2	NHBS ARV detection analysis [55] 95% CI	✓		
	2011	51.6	43.8	59.4	NHBS ARV detection analysis 95% CI	✓		
	2014	70.2	60.8	78.1	NHBS ARV detection analysis 95% CI			✓
Percentage of white HIV-positive MSM on ART	2008	61.1	38.6	79.7	NHBS ARV detection analysis [55] 95% CI	✓		
	2011		0	100	Numbers too small			
	2014		0	100	Numbers too small			
Percentage of diagnosed black MSM in care	2012-2013	63.5	56.7	70.3	Maryland DH[a] ± 5pp min-max for 2012-2013		✓	
	2014	60.3	55.3	65.3	Maryland DH[a] ± 5pp			✓

	2015	57.6	52.6	62.6	Maryland DH[a] ± 5pp			✓
	2016	57.1	52.1	62.1	Maryland DH[a] ± 5pp			✓
	2017	59.9	54.9	64.9	Maryland DH[a] ± 5pp			✓
Percentage of diagnosed white MSM in care	2012-2013	51.6	44.6	58.6	Maryland DH[a] ± 5pp min-max for 2012-2013		✓	
	2014	54.5	49.5	59.5	Maryland DH[a] ± 5pp			✓
	2015	49.7	44.7	54.7	Maryland DH[a] ± 5pp			✓
	2016	51.5	46.5	56.5	Maryland DH[a] ± 5pp			✓
	2017	56.0	51.0	61.0	Maryland DH[a] ± 5pp			✓
Percentage of diagnosed black MSM virally suppressed	2012	31.6	26.6	36.6	Maryland DH[b] ± 5pp		✓	
	2013	37.0	32.0	42.0	Maryland DH[b] ± 5pp		✓	
	2014	46.0	41.0	51.0	Maryland DH[b] ± 5pp			✓
	2015	44.9	39.9	49.9	Maryland DH[b] ± 5pp			✓
	2016	46.3	41.3	51.3	Maryland DH[a] ± 5pp			✓
	2017	50.9	45.9	55.9	Maryland DH[a] ± 5pp			✓
Percentage of diagnosed white MSM virally suppressed	2012	35.1	30.1	40.1	Maryland DH[b] ± 5pp		✓	
	2013	38.5	33.5	43.5	Maryland DH[b] ± 5pp		✓	
	2014	51.2	46.2	56.2	Maryland DH[b] ± 5pp			✓
	2015	51.4	46.4	56.4	Maryland DH[b] ± 5pp			✓
	2016	50.5	45.5	55.5	Maryland DH[a] ± 5pp			✓
	2017	55.0	50.0	60.0	Maryland DH[a] ± 5pp			✓
Percentage of MSM on ART virally suppressed	2010	85	75	90	National estimates for MSM[24, 56] range	✓	✓	

[a]definition of in care: percentage of those diagnosed with at least one CD4 test past 12 months

[b]definition of virally suppressed: percentage of those diagnosed with at least one viral load test last 12 months and most recent viral load <200 copies/ml

Illustrations for analysis

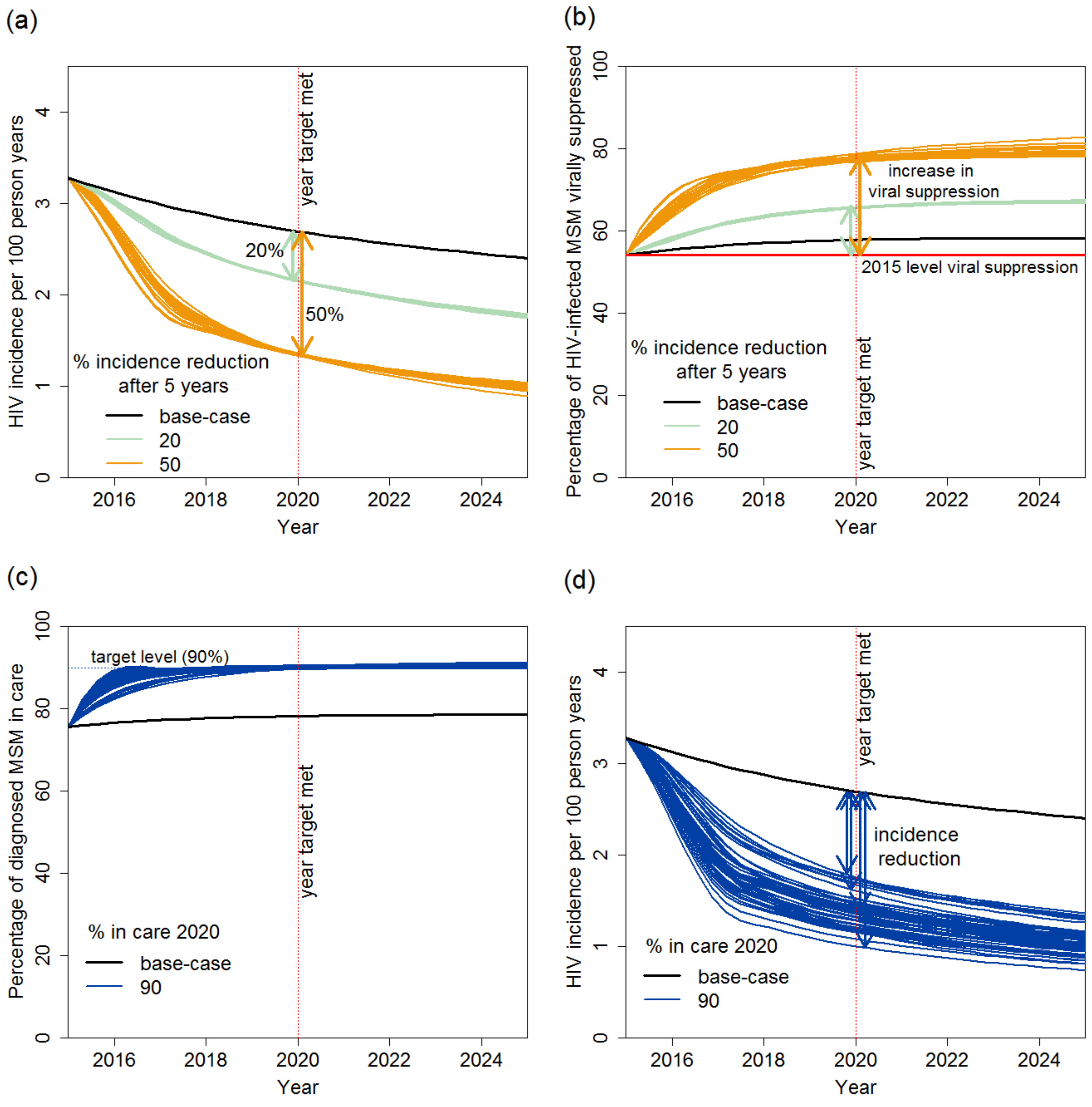

Fig S4. Illustration of calculations of targets and outcomes. Each line represents one model run using the same initial parameter combination, with different care continuum parameters from the start of 2016. The parameter combination chosen has similar incidence trends to the median incidence trend across all fits. The runs shown in (a) and (b) are selected runs which meet the 20% and 50% incidence reduction targets after 5 years – HIV incidence in 2020 in these runs in 2020 is (relatively) 20% and 50% lower than incidence in 2020 in the base-case scenario, where care continuum parameters remain unchanged from their 2015 values. The outcome measure is absolute increase in viral suppression levels in 2020 above 2015 levels; (a) shows HIV incidence, (b) shows levels of viral suppression. The runs shown in (c) and (d) are selected runs which meet the US continuum target of 90% of those diagnosed being in care in 2020. The outcome measure is relative reduction in HIV incidence in 2020 compared with the base-case scenario in 2020; (c) shows the percentage of those diagnosed who are in care, (d) shows HIV incidence. Red dashed vertical lines show the time-point at which the targets are met and outcome measures recorded. The solid red horizontal line shows initial level of viral suppression. The blue dashed horizontal line shows the level of the diagnosis target.

RESULTS

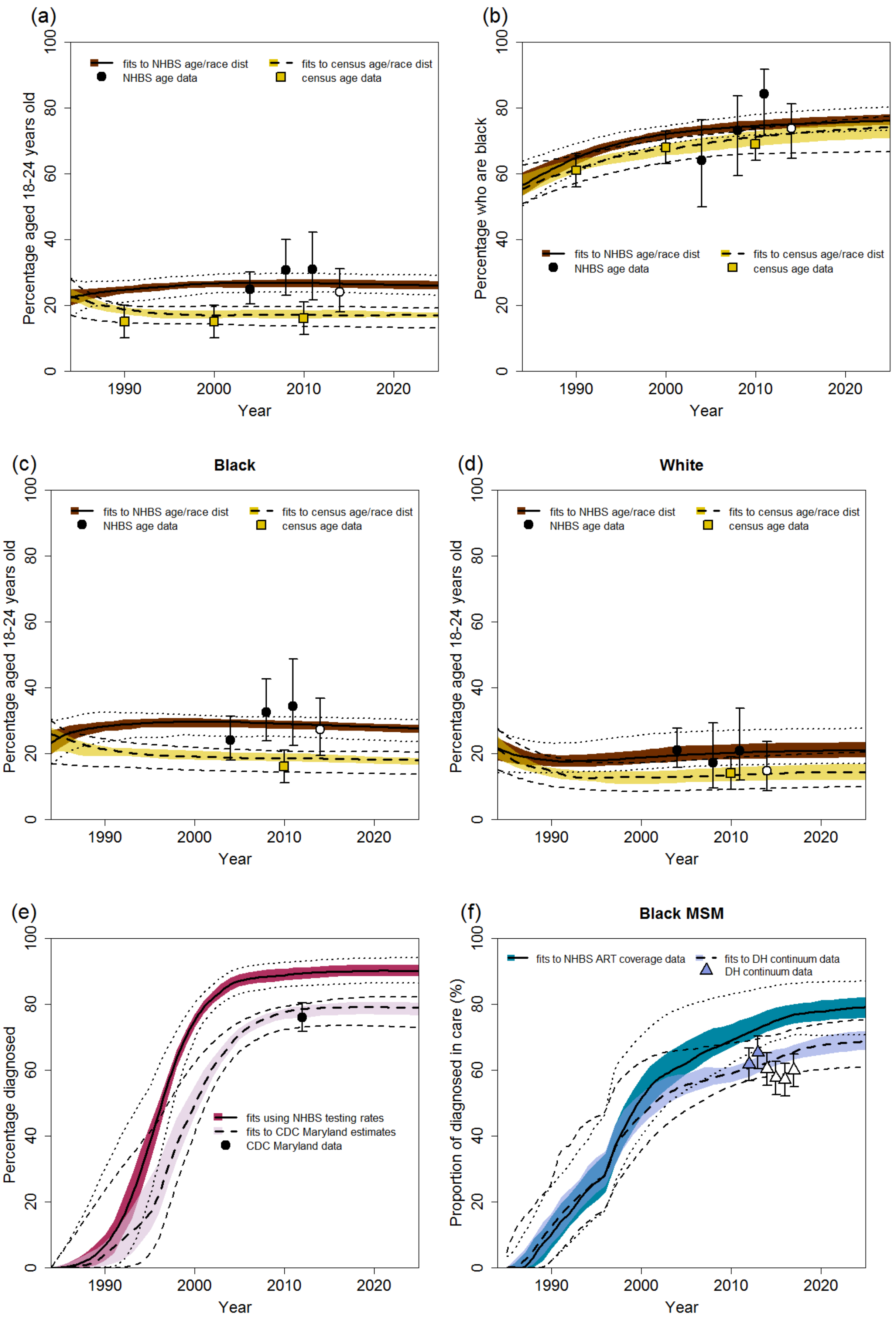

(a)
fits to NHBS age/race dist
NHBS age data
fits to census age/race dist
census age data
Percentage aged 18-24 years old
Year
(b)
Percentage who are black
Year
(c)
Black
Percentage aged 18-24 years old
Year
(d)
White
Percentage aged 18-24 years old
Year
(e)
Percentage diagnosed
fits using NHBS testing rates
fits to CDC Maryland estimates
CDC Maryland data
Year
(f)
Black MSM
fits to NHBS ART coverage data
fits to DH continuum data
DH continuum data
Proportion of diagnosed in care (%)
Year

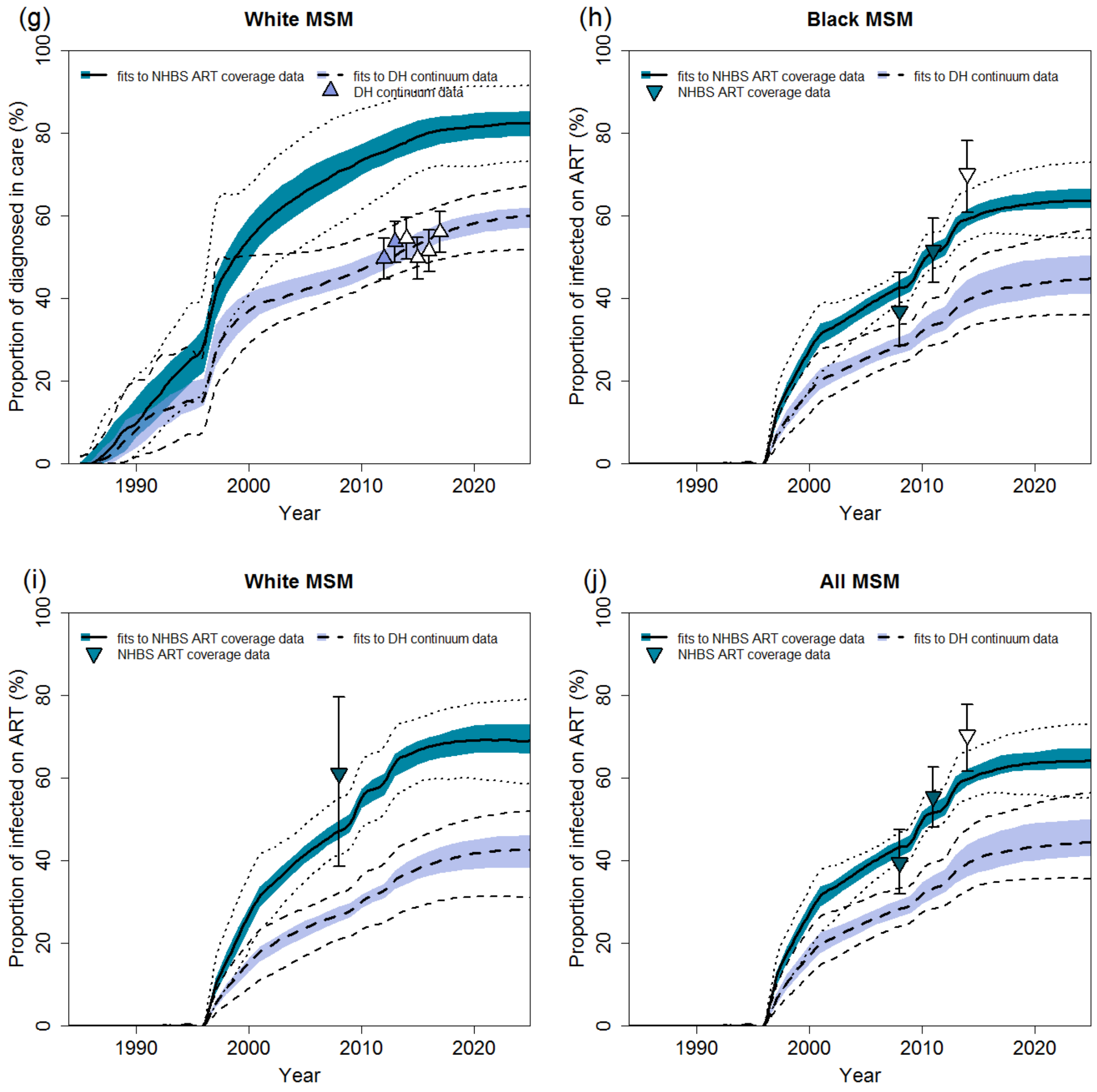
(g)
White MSM
fits to NHBS ART coverage data
fits to DH continuum data
DH continuum data
Proportion of diagnosed in care (%)
0
20
40
60
80
100
1990
2000
2010
2020
Year
(h)
Black MSM
fits to NHBS ART coverage data
fits to DH continuum data
NHBS ART coverage data
Proportion of infected on ART (%)
Year
(i)
White MSM
fits to NHBS ART coverage data
fits to DH continuum data
NHBS ART coverage data
Proportion of infected on ART (%)
Year
(j)
All MSM
fits to NHBS ART coverage data
fits to DH continuum data
NHBS ART coverage data
Proportion of infected on ART (%)
Year

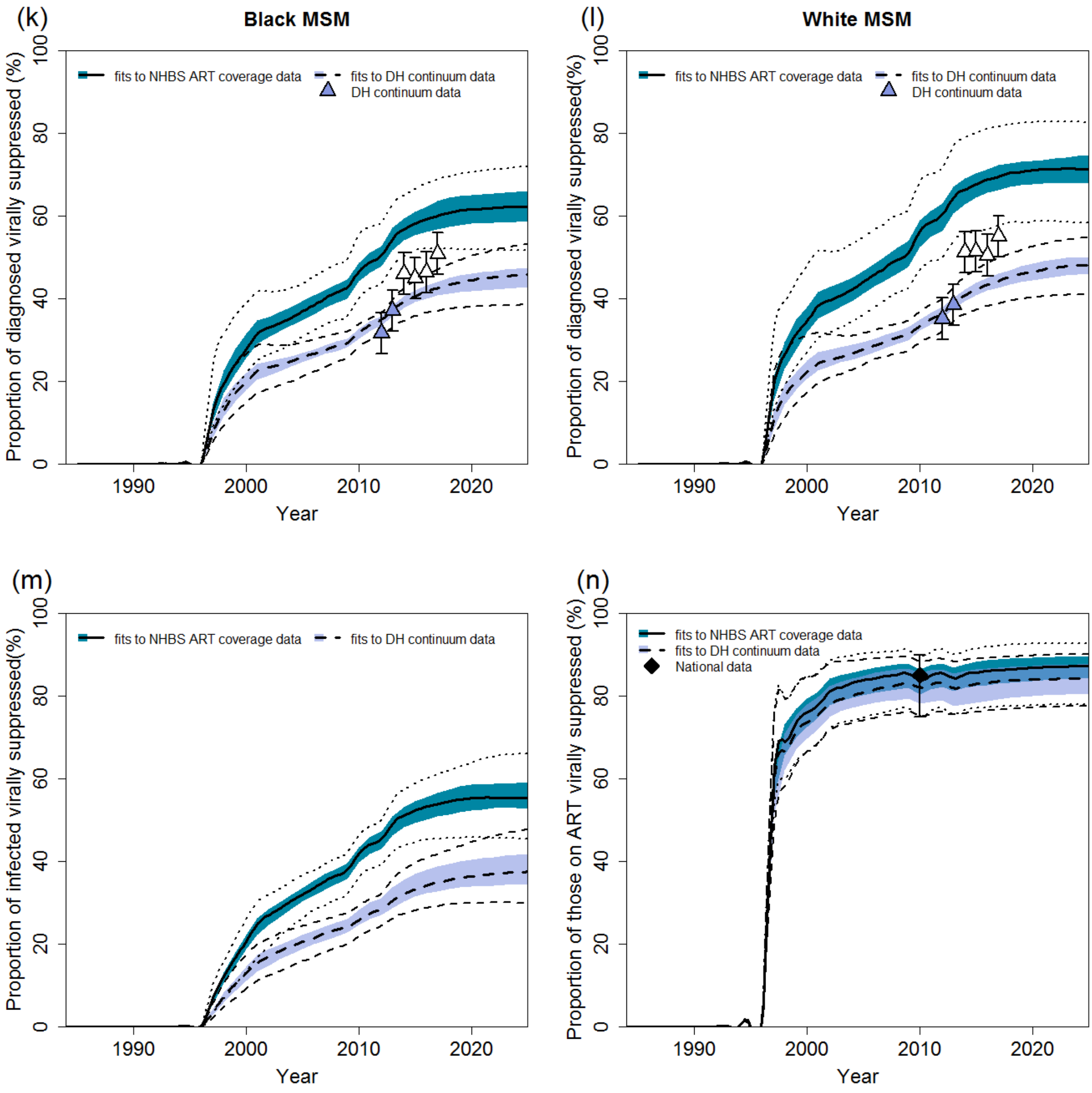

Fig S5. Outcomes for model fits: (a) percentage of all MSM aged 18-24 years old, (b) percentage of MSM who are black, (c) percentage of black MSM aged 18-24 years old, (d) percentage of white MSM aged 18-24 years old, (e) percentage of HIV-positive MSM who are diagnosed, (f) percentage of black diagnosed HIV-positive MSM who are linked into care, (g) percentage of white diagnosed HIV-positive MSM who are linked into care, (h) percentage of black HIV-positive MSM who are on ART, (i) percentage of white HIV-positive MSM who are on ART, (j) percentage of all HIV-positive MSM who are on ART, (k) percentage of black diagnosed HIV-positive MSM who are virally suppressed, (l) percentage of white diagnosed HIV-positive MSM who are virally suppressed, (m) percentage of HIV-positive MSM who are virally suppressed, (n) percentage of MSM on ART who are virally suppressed. Results show median (thick lines), 25th-75th percentile (dark shaded area), and 2.5th and 97.5th percentiles (lighter dashed lines) across model fits. Points and error bars show the mean and 95% CI for NHBS data (a,b,c,d,h, I, j), mean and ±5 percentage points for census data (a,b,c,d) and DH continuum data (f,g,k,l), mean and range for national data (n). Data prior to 2014 were used for model fitting. Data from 2014 and 2015 (white points) were not used for fitting but to validate model predictions. Number of fits under each assumption: demography fitting assumptions, NHBS age/race distribution (N=146), census age/race distribution (N=23); diagnosis fitting assumptions, NHBS HIV testing rate parameter (N=118), CDC estimates for Maryland (N=51); continuum fitting assumptions, NHBS ART coverage data (N= 101), DH continuum data (N= 68).

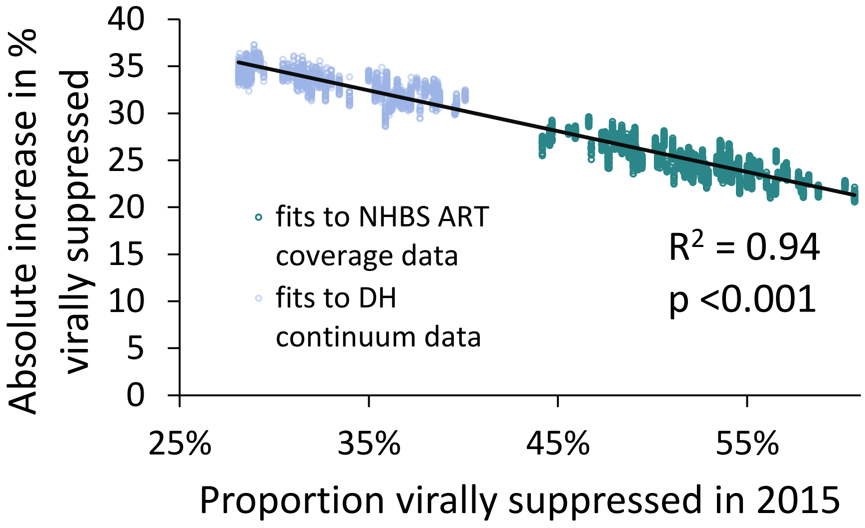

Fig S6. Required increase in viral suppression in 2020 above initial 2015 level needed to meet a 50% incidence reduction compared with the base-case scenario in 2020, for all runs across all fits, against initial level of viral suppression in 2015. Different colours show fits to different continuum assumptions: fits to NHBS ART coverage data in dark green, fits to DH continuum data in pale blue. Solid line shows the best fit regression line, R^2 value is for the linear regression model, p-value for the regression coefficient.

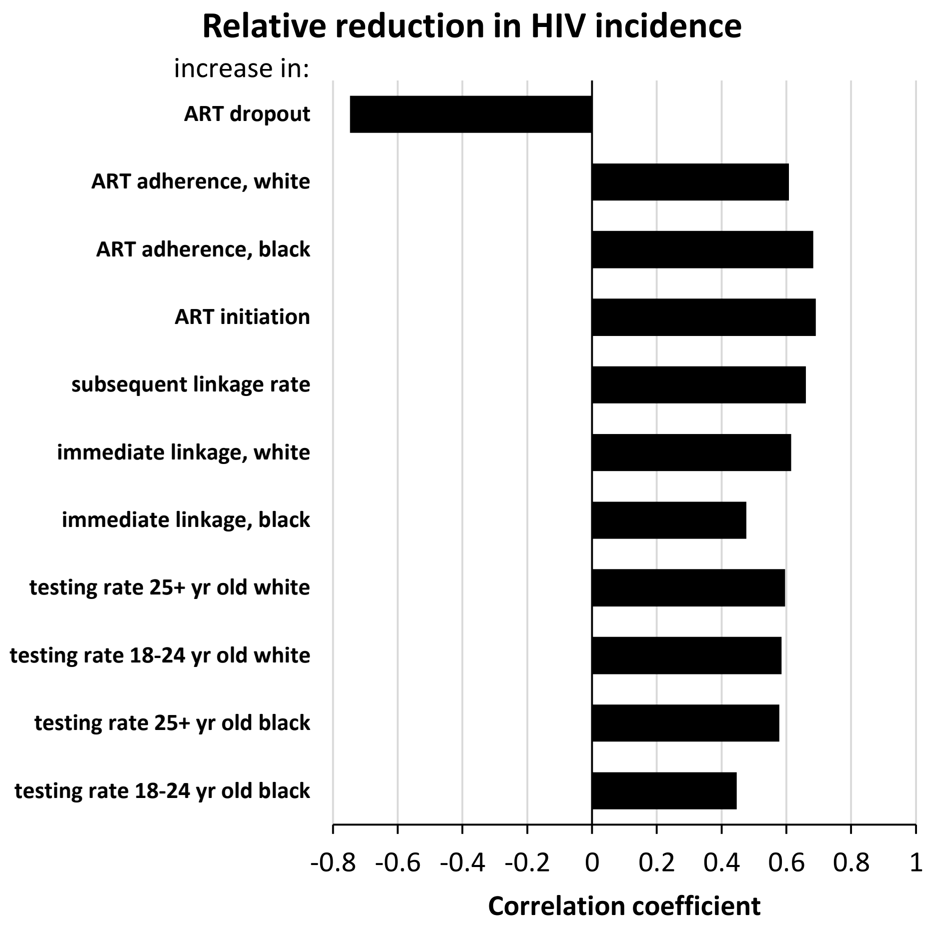

Fig S7. Correlations between the increase in care continuum parameters above 2015 levels (y-axis) and the relative reduction in HIV incidence after 5 years, for all runs.

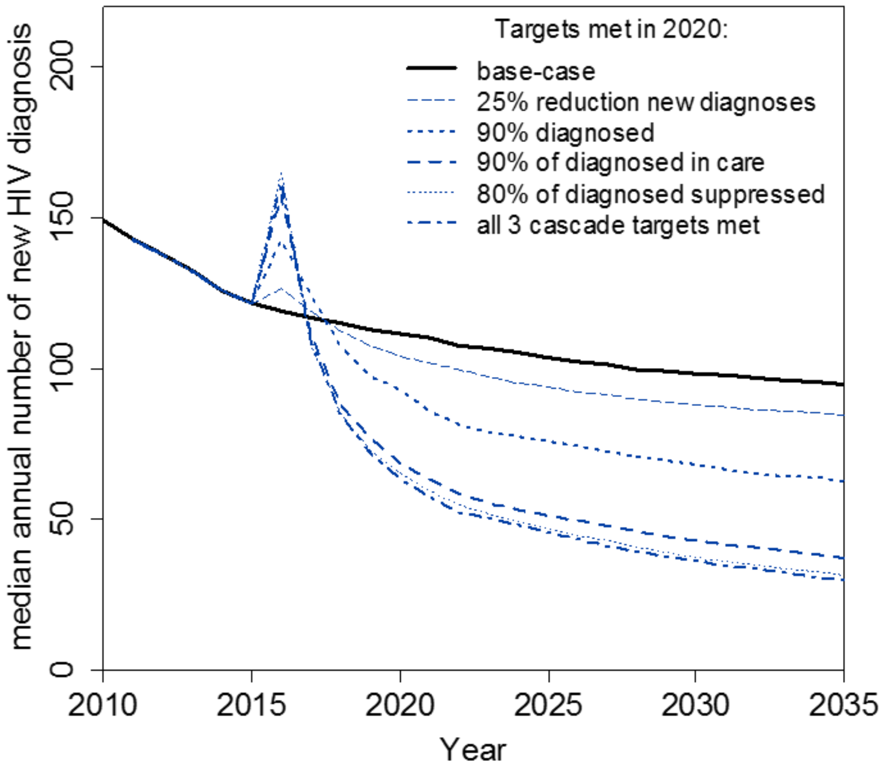

Fig S8. New HIV diagnoses over time under the base-case scenario and when different US targets are met in 2020. Plot shows the median value across means for up to 169 fits. Note the temporary increase in new HIV diagnoses following increases in HIV testing rates at the start of 2016, despite reductions in HIV incidence – this means that, in the short term, new HIV diagnoses do not reflect HIV incidence well.

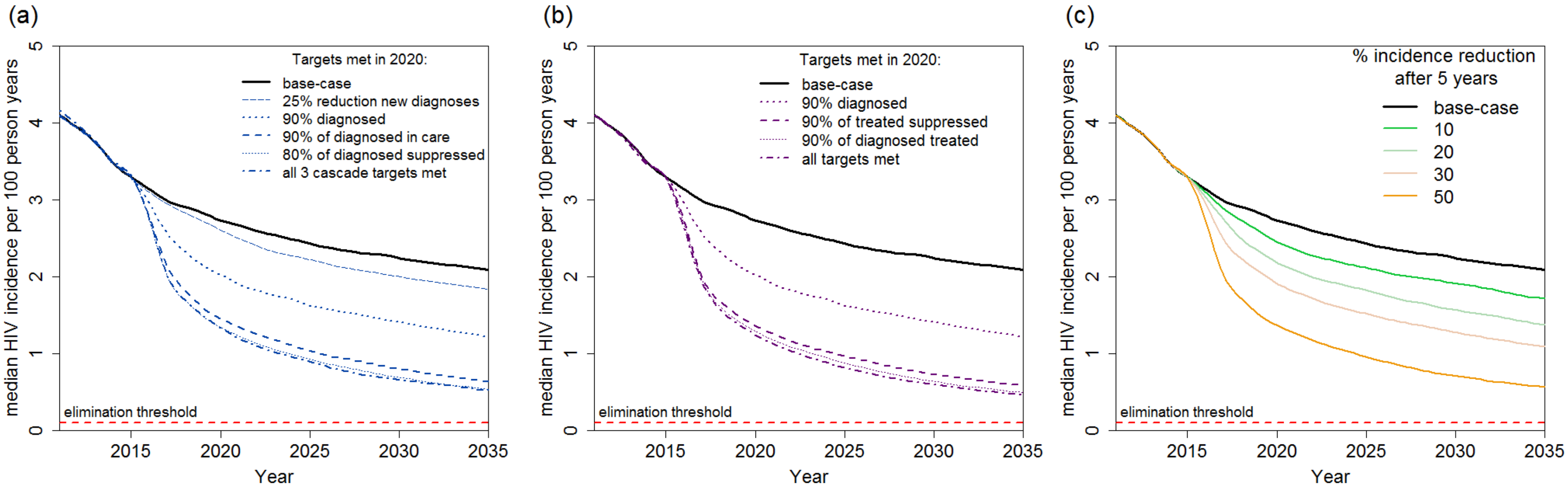

Fig S9. HIV incidence (new cases per 100 uninfected MSM per year) over time under the base-case scenario and when different targets are met. Plots show the median value across means for up to 169 fits when the following targets are met: (a) US targets met in 2020, (b) UNAIDS continuum targets met in 2020, (c) trial incidence reduction targets in comparison to the base-case scenario met after 5 years.. The dashed horizontal red line shows the HIV elimination threshold of 1 per 1000 person years. Note that some individual runs go below the elimination threshold – see tables S3a-S3cS1-S3.

Table S3a. Probability of reaching HIV elimination within 20 years when meeting different trial incidence reduction targets after different time periods. Number given are average percentage of runs in which HIV elimination is achieved within 20 years, across all fits meeting each target.

**Incidence reduction target:**	**10%**	**20%**	**30%**	**50%**
2 years	0	0	0.2%	5.9%
5 years	0	0	0	0.08%
10 years	0	0	0	0

Table S3b. Probability of reaching HIV elimination within 20 years when meeting different US continuum targets in 2020. Number given are average percentage of runs in which HIV elimination is achieved within 20 years, across all fits meeting each target. Note that when one target alone is met, the other targets may be exceeded or not met.

**US target:**	**90% of HIV-positive diagnosed**	**90% of diagnosed in care**	**80% of diagnosed virally suppressed**	**All 3 US targets together**
	0.03%	0.22%	0	0

Table S3c. Probability of reaching HIV elimination within 20 years when meeting different UNAIDS targets in 2020. Number given are average percentage of runs in which HIV elimination is achieved within 20 years, across all fits meeting each target. Note that when one target alone is met, the other targets may be exceeded or not met.

**UNAIDS target:**	**90% of HIV-positive diagnosed**	**90% of diagnosed treated**	**90% of treated virally suppressed**	**All 3 UNAIDS targets together**
	0.03%	0.24%	0.10%	0

REFERENCES

1. Phillips AN, Staszewski S, Weber R, Kirk O, Francioli P, Miller V, et al. HIV viral load response to antiretroviral therapy according to the baseline CD4 cell count and viral load. JAMA. 2001;286(20):2560-7.

2. CDC. WONDER online database. Available from: https://wonder.cdc.gov/.

3. Yeip R. Baltimore's Demographic Divide. Wall Street Journal. 2015.

4. Sullivan PS, Salazar L, Buchbinder S, Sanchez TH. Estimating the proportion of HIV transmissions from main sex partners among men who have sex with men in five US cities. AIDS. 2009;23(9):1153-62. doi: 10.1097/QAD.0b013e32832baa34.

5. Delaney KP, Rosenberg ES, Kramer MR, Waller LA, Sullivan PS. Optimizing human immunodeficiency virus testing interventions for men who have sex with men in the United States: a modeling study. Open Forum Infect Dis. 2015;2(4):ofv153. doi: 10.1093/ofid/ofv153.

6. Mitchell JW, Petroll AE. Patterns of HIV and sexually transmitted infection testing among men who have sex with men couples in the United States. Sex Transm Dis. 2012;39(11):871-6. doi: 10.1097/OLQ.0b013e3182649135.

7. Chmiel JS, Detels R, Kaslow RA, Van Raden M, Kingsley LA, Brookmeyer R. Factors associated with prevalent human immunodeficiency virus (HIV) infection in the Multicenter AIDS Cohort Study. Am J Epidemiol. 1987;126(4):568-77.

8. Cori A, Pickles M, van Sighem A, Gras L, Bezemer D, Reiss P, et al. CD4+ cell dynamics in untreated HIV-1 infection: overall rates, and effects of age, viral load, sex and calendar time. AIDS. 2015;29(18):2435-46. doi: 10.1097/qad.0000000000000854.

9. Dunn D, Woodburn P, Duong T, Peto J, Phillips A, Gibb D, et al. Current CD4 cell count and the short-term risk of AIDS and death before the availability of effective

antiretroviral therapy in HIV-positive children and adults. J Infect Dis. 2008;197(3):398-404. doi: 10.1086/524686.

10. Antiretroviral Therapy Cohort Collaboration. Importance of baseline prognostic factors with increasing time since initiation of highly active antiretroviral therapy: collaborative analysis of cohorts of HIV-1-infected patients. J Acquir Immune Defic Syndr. 2007;46(5):607-15. doi: 10.1097/QAI.0b013e31815b7dba.

11. Hollingsworth TD, Anderson RM, Fraser C. HIV-1 transmission, by stage of infection. J Infect Dis. 2008;198(5):687-93. doi: 10.1086/590501.

12. Donnell D, Baeten JM, Kiarie J, Thomas KK, Stevens W, Cohen CR, et al. Heterosexual HIV-1 transmission after initiation of antiretroviral therapy: a prospective cohort analysis. Lancet. 2010;375(9731):2092-8. doi: 10.1016/s0140-6736(10)60705-2.

13. Baggaley RF, White RG, Boily MC. HIV transmission risk through anal intercourse: systematic review, meta-analysis and implications for HIV prevention. Int J Epidemiol. 2010;39(4):1048-63. doi: 10.1093/ije/dyq057.

14. Jin F, Jansson J, Law M, Prestage GP, Zablotska I, Imrie JC, et al. Per-contact probability of HIV transmission in homosexual men in Sydney in the era of HAART. AIDS. 2010;24(6):907-13. doi: 10.1097/QAD.0b013e3283372d90.

15. Rodger AJ, Cambiano V, Bruun T, Vernazza P, Collins S, van Lunzen J, et al. Sexual activity without condoms and risk of HIV transmission in serodifferent couples when the HIV-positive partner is using suppressive antiretroviral therapy. JAMA. 2016;316(2):171-81. doi: 10.1001/jama.2016.5148.

16. Blaser N, Wettstein C, Estill J, Vizcaya LS, Wandeler G, Egger M, et al. Impact of viral load and the duration of primary infection on HIV transmission: systematic review and meta-analysis. AIDS. 2014;28(7):1021-9. doi: 10.1097/qad.0000000000000135.

17. Smith DK, Herbst JH, Zhang X, Rose CE. Condom effectiveness for HIV prevention by consistency of use among men who have sex with men in the United States. J Acquir Immune Defic Syndr. 2015;68(3):337-44. doi: 10.1097/qai.0000000000000461.

18. Mills E, Cooper C, Anema A, Guyatt G. Male circumcision for the prevention of heterosexually acquired HIV infection: a meta-analysis of randomized trials involving 11,050 men. HIV Med. 2008;9(6):332-5. doi: 10.1111/j.1468-1293.2008.00596.x.

19. Anderson JE, Carey JW, Taveras S. HIV testing among the general US population and persons at increased risk: information from national surveys, 1987-1996. Am J Public Health. 2000;90(7):1089-95.

20. Hoots BE, Finlayson TJ, Wejnert C, Paz-Bailey G. Early linkage to HIV care and antiretroviral treatment among men who have sex with men - 20 cities, United States, 2008 and 2011. PLoS ONE. 2015;10(7):e0132962. doi: 10.1371/journal.pone.0132962.

21. Center for HIV Surveillance Epidemiology and Evaluation Maryland Department of Health and Mental Hygiene. Baltimore City annual HIV epidemiological profile 2013. Baltimore, MD. : 2015.

22. Center for HIV Surveillance Epidemiology and Evaluation Maryland Department of Health and Mental Hygiene. 2012 Baltimore City annual HIV epidemiological profile. Baltimore, MD. : 2015.

23. Center for HIV Surveillance Epidemiology and Evaluation Maryland Department of Health and Mental Hygiene. Baltimore City annual HIV epidemiological profile 2015 Baltimore, MD.2016.

24. Singh S, Bradley H, Hu X, Skarbinski J, Hall HI, Lansky A. Men living with diagnosed HIV who have sex with men: progress along the continuum of HIV care--United States, 2010. MMWR. 2014;63(38):829-33.

25. Centers for disease control and prevention. Reported CD4+ T-lymphocyte results for adults and adolescents with HIV/AIDS - 33 states, 2005. HIV/AIDS Surveillance Report. 2005;11(2).

26. Centers for Disease Control and Prevention. Reported CD4+ T-lymphocyte and viral load results for adults and adolesecents with HIV infection - 37 states, 2005-2007. HIV Surveillance Supplemental Report. 2010;16(1).

27. Tedaldi EM, Richardson JT, Debes R, Young B, Chmiel JS, Durham MD, et al. Retention in care within 1 year of initial HIV care visit in a multisite US cohort: who's in and who's out? J Int Assoc Provid AIDS Care. 2014;13(3):232-41. doi: 10.1177/2325957413514631.

28. Li X, Margolick JB, Conover CS, Badri S, Riddler SA, Witt MD, et al. Interruption and discontinuation of highly active antiretroviral therapy in the multicenter AIDS cohort study. J Acquir Immune Defic Syndr. 2005;38(3):320-8.

29. Novak RM, Hart RL, Chmiel JS, Brooks JT, Buchacz K. Disparities in initiation of combination antiretroviral treatment and in virologic suppression among patients in the HIV Outpatient Study (HOPS), 2000-2013. J Acquir Immune Defic Syndr. 2015. doi: 10.1097/qai.0000000000000652.

30. Weintrob AC, Grandits GA, Agan BK, Ganesan A, Landrum ML, Crum-Cianflone NF, et al. Virologic response differences between African Americans and European Americans initiating highly active antiretroviral therapy with equal access to care. J Acquir Immune Defic Syndr. 2009;52(5):574-80. doi: 10.1097/QAI.0b013e3181b98537.

31. Krishnan S, Wu K, Smurzynski M, Bosch RJ, Benson CA, Collier AC, et al. Incidence rate of and factors associated with loss to follow-up in a longitudinal cohort of antiretroviral-treated HIV-positive persons: an AIDS Clinical Trials Group (ACTG)

Longitudinal Linked Randomized Trials (ALLRT) analysis. HIV Clin Trials. 2011;12(4):190-200. doi: 10.1310/hct1204-190.

32. Purcell DW, Johnson CH, Lansky A, Prejean J, Stein R, Denning P, et al. Estimating the population size of men who have sex with men in the United States to obtain HIV and syphilis rates. Open AIDS J. 2012;6:98-107. doi: 10.2174/1874613601206010098.

33. Easterbrook PJ, Chmiel JS, Hoover DR, Saah AJ, Kaslow RA, Kingsley LA, et al. Racial and ethnic differences in human immunodeficiency virus type 1 (HIV-1) seroprevalence among homosexual and bisexual men. The Multicenter AIDS Cohort Study. Am J Epidemiol. 1993;138(6):415-29.

34. Maulsby C, Jain K, Sifakis F, German D, Flynn CP, Holtgrave D. Individual-level and partner-level predictors of newly diagnosed HIV infection among black and white men who have sex with men in Baltimore, MD. AIDS Behav. 2014. doi: 10.1007/s10461-014-0861-5.

35. Boily MC, Baggaley RF, Wang L, Masse B, White RG, Hayes RJ, et al. Heterosexual risk of HIV-1 infection per sexual act: systematic review and meta-analysis of observational studies. Lancet Infect Dis. 2009;9(2):118-29. doi: 10.1016/s1473-3099(09)70021-0.

36. Egger M, May M, Chene G, Phillips AN, Ledergerber B, Dabis F, et al. Prognosis of HIV-1-infected patients starting highly active antiretroviral therapy: a collaborative analysis of prospective studies. Lancet. 2002;360(9327):119-29.

37. UK Office for National Statistics. Deaths: age sex. England and Wales [table 6.1]. Population Trends. 2006;126:49.

38. Michigan Department of Community Health. Adult and Adolescent Spectrum of Disease Project in Michigan Summary Report 1990-2003.

39. Guiguet M, Porter K, Phillips A, Costagliola D, Babiker A. Clinical progression rates by CD4 cell category before and after the initiation of combination antiretroviral therapy (cART). Open AIDS J. 2008;2:3-9. doi: 10.2174/1874613600802010003.

40. Mocroft A, Furrer HJ, Miro JM, Reiss P, Mussini C, Kirk O, et al. The incidence of AIDS-defining illnesses at a current CD4 count >/= 200 cells/muL in the post-combination antiretroviral therapy era. Clin Infect Dis. 2013;57(7):1038-47. doi: 10.1093/cid/cit423.

41. Mellors JW, Munoz A, Giorgi JV, Margolick JB, Tassoni CJ, Gupta P, et al. Plasma viral load and CD4+ lymphocytes as prognostic markers of HIV-1 infection. Ann Intern Med. 1997;126(12):946-54.

42. Herbeck JT, Gottlieb GS, Li X, Hu Z, Detels R, Phair J, et al. Lack of evidence for changing virulence of HIV-1 in North America. PLoS ONE. 2008;3(2):e1525. doi: 10.1371/journal.pone.0001525.

43. Drake AL, Kinuthia J, Matemo D, McClelland RS, Richardson BA, Overbaugh J, et al. Virologic and immunologic response following antiretroviral therapy initiation among pregnant and postpartum women with acute HIV-1 infection [abstract #MOPDB0101]. International AIDS conference; Melbourne, Australia; 2014.

44. Donnell D, Baeten JM, Kiarie J, Thomas KK, Stevens W, Cohen CR, et al. Heterosexual HIV-1 transmission after initiation of antiretroviral therapy: a prospective cohort analysis. Lancet. 2010;375(9731):2092-8.

45. Hallett TB, Baeten JM, Heffron R, Barnabas R, de Bruyn G, Cremin Í, et al. Optimal uses of antiretrovirals for prevention in HIV-1 serodiscordant heterosexual couples in South Africa: a modelling study. PLoS Med. 2011;8(11):e1001123. doi: 10.1371/journal.pmed.1001123.

46. Moore RD, Keruly JC, Bartlett JG. Improvement in the health of HIV-positive persons in care: reducing disparities. Clin Infect Dis. 2012;55(9):1242-51. doi: 10.1093/cid/cis654.

47. Rebeiro P, Althoff KN, Buchacz K, Gill J, Horberg M, Krentz H, et al. Retention among North American HIV-positive persons in clinical care, 2000-2008. J Acquir Immune Defic Syndr. 2013;62(3):356-62. doi: 10.1097/QAI.0b013e31827f578a.

48. Althoff KN, Rebeiro P, Brooks JT, Buchacz K, Gebo K, Martin J, et al. Disparities in the quality of HIV care when using US Department of Health and Human Services indicators. Clin Infect Dis. 2014;58(8):1185-9. doi: 10.1093/cid/ciu044.

49. Cohen MS, Chen YQ, McCauley M, Gamble T, Hosseinipour MC, Kumarasamy N, et al. Antiretroviral therapy for the prevention of HIV-1 transmission. N Engl J Med. 2016. doi: 10.1056/NEJMoa1600693.

50. Howe CJ, Cole SR, Napravnik S, Eron JJ. Enrollment, retention, and visit attendance in the University of North Carolina Center for AIDS Research HIV clinical cohort, 2001-2007. AIDS Res Hum Retroviruses. 2010;26(8):875-81. doi: 10.1089/aid.2009.0282.

51. Dasgupta S, Oster AM, Li J, Hall HI. Disparities in consistent retention in HIV care - 11 states and the district of Columbia, 2011-2013. MMWR Morb Mortal Wkly Rep. 2016;65(4):77-82. doi: 10.15585/mmwr.mm6504a2.

52. Robertson M, Laraque F, Mavronicolas H, Braunstein S, Torian L. Linkage and retention in care and the time to HIV viral suppression and viral rebound - New York City. AIDS Care. 2015;27(2):260-7. doi: 10.1080/09540121.2014.959463.

53. Palella FJ, Jr., Delaney KM, Moorman AC, Loveless MO, Fuhrer J, Satten GA, et al. Declining morbidity and mortality among patients with advanced human immunodeficiency virus infection. HIV Outpatient Study Investigators. N Engl J Med. 1998;338(13):853-60. doi: 10.1056/nejm199803263381301.

54. Hall HI, An Q, Tang T, Song R, Chen M, Green T, et al. Prevalence of diagnosed and undiagnosed HIV infection - United States, 2008-2012. MMWR. 2015;64(24):657-62.

55. German D, Shearer K, Park JN, Flynn C, Latkin C, Laeyendecker O, et al. Factors associated with misreporting HIV status among MSM from Baltimore [abstract 906]. CROI; Boston, USA; 2016.

56. Hall HI, Frazier EL, Rhodes P, Holtgrave DR, Furlow-Parmley C, Tang T, et al. Differences in human immunodeficiency virus care and treatment among subpopulations in the United States. JAMA Intern Med. 2013;173(14):1337-44. doi: 10.1001/jamainternmed.2013.6841.
